# Microbiome-mitochondria crosstalk in neuro-oncology: molecular mechanisms, immunometabolic reprogramming, and therapeutic opportunities

**DOI:** 10.3389/frmbi.2026.1983174

**Published:** 2026-10-21

**Authors:** Debabrata Dash, Muskan Rajak, Priyanka Manothiya, Raj Kumar Koiri

**Affiliations:** Biochemistry Laboratory, Department of Zoology, Dr. Harisingh Gour Vishwavidyalaya (A Central University), Sagar, India

**Keywords:** glioblastoma, gut microbiome, gut-brain axis, microbiome-mitochondria crosstalk, tumor immunometabolism

## Abstract

Although the gut microbiome is increasingly recognized as an important regulator of metabolic, immune, and neurophysiological functions, and mitochondrial dysfunction is considered a hallmark of metabolic flexibility, oxidative stress, and resistance to therapy in cancers, the role of microbiome-derived cues and mitochondrial processes in brain tumors remains poorly understood. The Microbiome-Gut-Brain-Mitochondria Axis in Neuro-Oncology provides a framework for understanding metabolism and immunity in neuro-oncology by highlighting this axis and emphasizing its role in glioblastoma. Metabolites produced by the microbiome, including short-chain fatty acids, bile acid metabolites, tryptophan metabolites, and other microbial compounds, have been shown to affect the immune system, barrier function, cellular metabolism, and neural signaling. Mitochondria are capable of functioning as intracellular communication mechanisms that will help to decipher those signals in order to create energy, ROS, calcium communication, biogenesis of mitochondria, quality control, and apoptosis. These processes may contribute to metabolic plasticity in glioblastomas, including glycolytic, oxidative phosphorylation, and amino- and lipid-metabolic adaptations, mitochondrial transfer, adaptation to hypoxia, tumor cell stemness, and immune suppression in the tumor microenvironment. The article also discusses the relationships between microbiome-derived cues, mitochondria, and tumor-associated immune populations. Microbiome-derived and mitochondrial biomarkers, combined with multi-omics approaches, single-cell technologies, spatial biology, artificial intelligence, and new experimental models, may help to identify relevant microbiome-mitochondria signatures. Modulating the microbiome, mitochondria, and combination therapies appears promising for glioblastomas. However, the causative link between microbiome-derived mitochondrial mechanisms and human brain tumors remains unproven. The necessity of prospective and mechanistic studies of the microbiome, mitochondria, metabolism, immunity, and clinical aspects arises.

## Graphical Abstract

**Figure:**
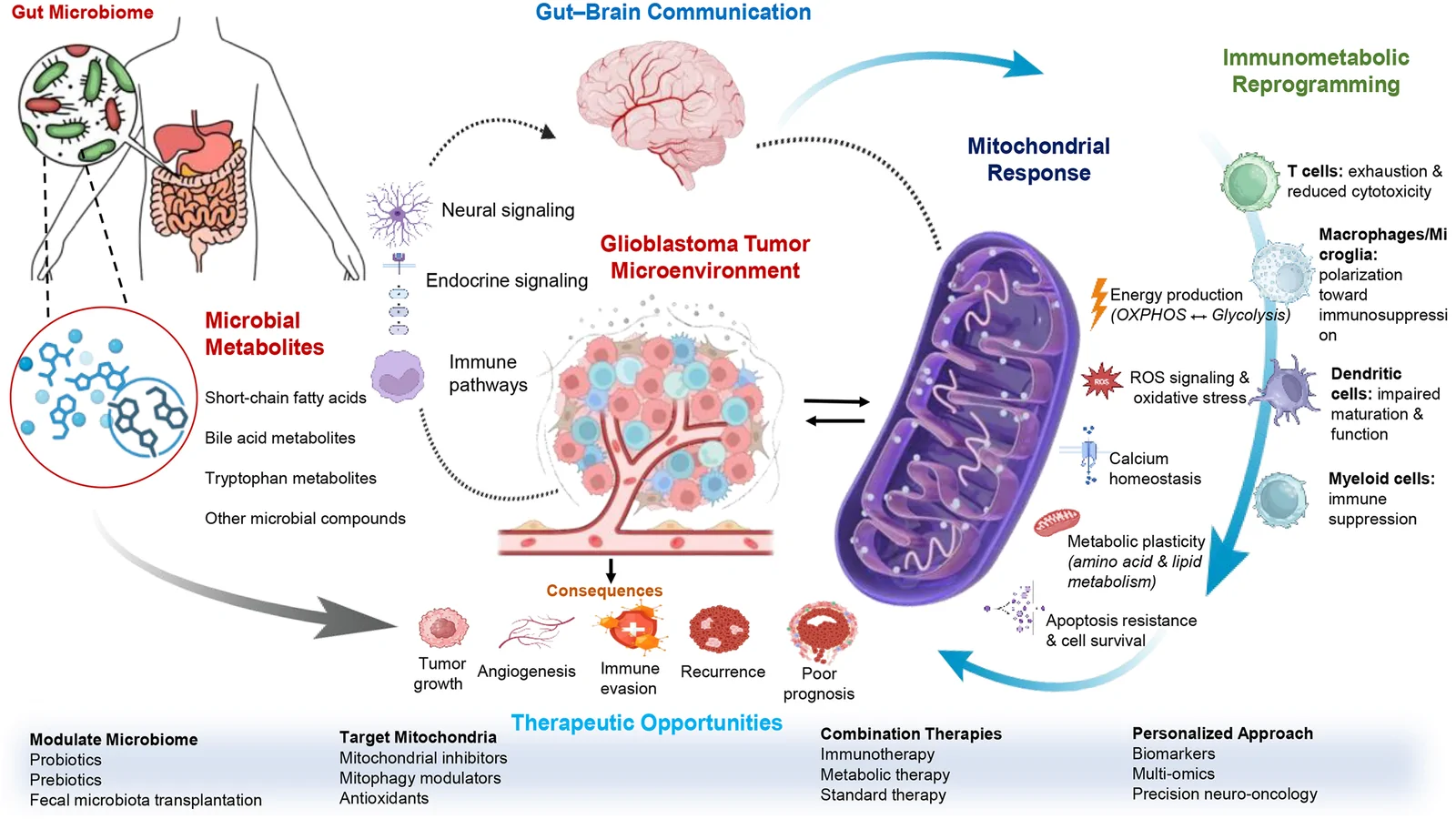

## 1 Introduction

Cancer is a dynamic ecosystem in which cancer cells interact with the TME, including immune cells, stromal cells, blood vessels, and nerves (Hausman, 2019; De Visser and Joyce, 2023). Since the autonomic nervous system controls the homeostatic response, any neurogenic stimuli within the TME can cause the immune cells to avoid immune reactions and lead to tumor development (Gibbons, 2019; Khanmammadova et al., 2023). At the same time, brain tumors signal to the peripheral and central nervous systems through various cytokines, adipokines, neurotrophins, and neuronal signals, creating a bidirectional interaction between tumors and the nervous systems (Magnon and Hondermarck, 2023). This issue is especially important for neuro-oncology since brain tumors are characterized by great biological heterogeneity. Thus, pediatric brain tumors include low-grade glioma, ependymoma, high-grade glioma, medulloblastoma, ganglioglioma, craniopharyngioma, and atypical teratoid/rhabdoid tumor (Petralia et al., 2020). Glioblastoma (GBM) is the most frequent and aggressive primary brain malignancy among adults despite the recent developments in multimodal treatment of GBM, which is often followed by local relapse (Zhao et al., 2023a). Brain tumors such as spinal cord astrocytoma are relatively rare (Pang et al., 2024). On the other hand, medulloblastomas are categorized based on molecular characteristics into WNT, SHH, Group 3, and Group 4 (Sharma et al., 2019). Metastasis in the brain is common among brain tumors and is an important part of neuro-oncology (Lamba et al., 2021).

Gut microbiome is thought of as a systemic modulator of the immunological, metabolic, structural, and neurologic functions due to the production of microbial, immunologic, hormonal, and neural signaling molecules into distant organ systems (Adak and Khan, 2018; Verma et al., 2024). Microbiomes play an essential role in the generation of SCFAs, enhancement of the integrity of intestinal barrier, maintenance of gut physiology and homeostatic balance between metabolism and immunity of the host. Microflora in the gut can regulate the production of neurotransmitters such as dopamine, serotonin, glutamate, and GABA (Qu et al., 2024). Although most of the studies on the axis gut-brain have been done to show its relevance in neurological and neuropsychiatric conditions, the systemic microbial signals could also play a role in inflammation and brain tumor metabolism.

It has been suggested that mitochondria might be a molecular mediator of these signals on brain tumor biology. In addition to energy production through oxidative phosphorylation, mitochondria regulate intracellular calcium homeostasis, reactive oxygen species (ROS), redox homeostasis, innate immune responses, apoptosis, and cell metabolism (Dache and Thierry, 2023). Mutations and alterations of the function of mitochondrial proteins and metabolism as well as the nuclear genome part of tricarboxylic acid (TCA) cycle contribute to cancer development (Zhang et al., 2024). Due to the critical role of mitochondria in metabolism and cell survival, they became a target for cancer treatments (Zong et al., 2016). GBM uses metabolic plasticity to use different pathways such as glycolysis, oxidative phosphorylation, amino-acid metabolism, and lipid metabolism depending on the environmental and cell-specific conditions (Garofano et al., 2021). Transfer of mitochondria horizontally from astrocytes to GBM cells is also thought to be a mechanism which influences adaptation and tumor cell development (Watson et al., 2023).

This supports a recently emerging gut-brain-mitochondria axis in neuro-oncology. Dysfunction and altered mitochondrial dynamics are observed in neurological diseases in which the gut-brain axis contributes to pathology (Borbolis et al., 2023). Therefore, metabolites and other signals from the microbiome can modulate mitochondrial bioenergetics, redox homeostasis, calcium signaling, mitochondrial dynamics, and cellular stress response, affecting tumor metabolism and the immune state of the brain tumor microenvironment. Mitochondrial alterations may lead to the release of metabolic and inflammatory factors that regulate the interplay with the microbiome. Such a two-way regulation mechanism might influence the survival, metabolic plasticity, stemness, immunosuppression, invasion, treatment resistance, and recurrence of tumor cells.

Nevertheless, the link between the microbiome and mitochondria in neuro-oncology is still a developing idea and not yet a proven causative effect. The absence of any evidence on the ability of microbiome alterations to induce certain mitochondrial phenotypes in human brain tumors leaves no space for conjectures. Most of the current rationale for the interaction of microbiome and mitochondria in neuro-oncology is based on complementary data from studies of microbiome-host interactions, neurological disorders, other cancers, and *in vitro* experiments. Thus, instead of suggesting a pre-existing microbiome-mitochondria axis, the key purpose of this review is to provide evidence for the biological plausibility of such an interaction, differentiate direct evidence of GBM involvement from mechanistic speculation, highlight knowledge gaps regarding specific links between microbial metabolites and mitochondrial phenotypes, and outline approaches to establish causality and translatability.

Other reviews have covered the connections between the gut microbiome and glioblastoma development, progression, immune response, and treatment, and yet others have been mainly concerned with mitochondrial metabolism and potential therapeutic targeting of mitochondria in GBM (Ganesh et al., 2025; Toumazi et al., 2025; Liang et al., 2025). In contrast, this review emphasizes the central role of the microbiome-mitochondria interface in GBM pathobiology and connects two previously unconnected domains into a single mechanistic framework. We specifically focus on how metabolites and signals produced by the microbiome could converge on mitochondrial bioenergetics, redox regulation, calcium signaling, mitochondrial dynamics, and mitochondrial quality control, and interact with metabolic reprogramming of tumor cells and immune cells. In addition, we relate the above-mentioned processes to tumor proliferation, invasion, stemness, adaptation to hypoxic conditions, therapy resistance, relapse, and diagnostic/therapeutic biomarkers in GBM. Rather than treating the microbiome and mitochondria as independent regulators of GBM biology, we aim to evaluate their potential interactions and outline the evidence that would allow us to assess the nature of this connection: causal or associative.

## 2 The gut-brain-microbiome axis in neuro-oncology

Gut-brain axis is defined as a two-way interaction where the gut microbes, gut, immune system, endocrine communication, and nerves constantly influence each other. Contrary to a neural circuit, the system includes both microbial signaling, immunological signaling, neural signaling, and endocrine communication to control the physiological mechanisms of the host including neurodevelopment and brain physiology (Wang et al., 2023). This term has become highly important in neuro-oncology due to the impact of the gut microbiome changes on the systems of brain tumor biology.

### 2.1 Evolution of the gut-brain axis concept in brain tumors

The relationship of interactions between the gastrointestinal system and the brain has evolved greatly throughout the past few centuries. Physiological and clinical investigations revealed associations between digestion and behavioral and psychological conditions. The biological mechanisms of the interactions were thoroughly researched during the 20th century. Thanks to the advances in microbiome research and molecular techniques, the field of gut-brain interactions has been advanced through the change of perspective from a single neural communication channel to a multifactorial network consisting of microbial, metabolic, immune, endocrine, and neuronal communication (Lewandowska-Pietruszka et al., 2022).

Therefore, nowadays the term gut-brain axis describes a bi-directional interaction between the gastrointestinal tract, microbiome, and central nervous system (CNS). Communication channels include microbial metabolites, immune mediators, endocrine signals, neural pathways, and changes in epithelial and blood-brain barrier (BBB) functions. Such communication channels can potentially be involved in influencing the development of tumors of the brain by systemic changes in the gut environment. Modern data suggest that the dysfunction of gut microbiota can lead to systemic immune suppression, alterations in BBB permeability, and inflammatory state which can contribute to the biological environment for gliomas (Scuderi et al., 2025). Thus, information about the effects of gut microbiome on neural activity and behavior expands the classical definition of the gut-brain axis to non-neurological conditions (Miller, 2018).

It is important to note that the relevance of this axis for neuro-oncology does not mean that gut dysbiosis is the causative factor of brain tumors. Instead, it suggests a systemic regulatory layer that may alter tumor-associated immunity, metabolism, barrier function, and therapy. This point matters because much of the current evidence is associative and only indirectly demonstrates mechanisms in human brain tumors.

### 2.2 Gut microbiota as a systemic regulator of neuro-oncology

One major way the gut microbiome influences distant tissues is by producing and transforming bioactive metabolites. Microbiome-derived molecules such as short-chain fatty acids (SCFAs), bile acids, branched-chain amino acids, trimethylamine N-oxide (TMAO), tryptophan metabolites, and indoles and their derivatives can enter systemic circulation and act as signaling molecules that alter the host’s metabolic and immune responses (Agus et al., 2020). Therefore, these molecules could function as mediators connecting the action of intestinal microbes to other tissues. Indeed, the metabolites derived from the microbiome have recently become known as the remote regulators of the tumor immune response in various tissues (Liu H et al., 2025). Another aspect of systemic communication is the connection between the microbiome and host redox biology. ROS play important physiological roles in signal transduction. However, the excess of the ROS production above the organism’s antioxidative capacity is harmful. The redox imbalance may affect both the physiology of intestinal epithelium and the microbial community composition. In turn, the gut microbes and their metabolites can modify the mitochondrial homeostasis and ROS production of the host. This connection is particularly significant in terms of cancer studies since the redox balance controls inflammatory responses, metabolism, cell survival, and treatment resistance.

The regulation of the immune system by the gut microbiota is another way how the changes in the microbial populations may affect neuro-oncology. Different microbial populations in the intestines control the immune responses by modulating the populations of dendritic cells, macrophages, natural killer cells, CD4+ and CD8+ T lymphocytes, regulatory T lymphocytes (Tregs), and myeloid-derived suppressor cells (MDSCs). Therefore, the immune responses to the tumor can change, and the composition and functional status of the tumor-associated immune compartment can be altered (Guo et al., 2023). More broadly, the structure of the gut microbiome is associated with differences in antitumor immunity and the efficiency of treatments, including immune checkpoint inhibitors (ICIs) targeting programmed cell death protein 1 (PD-1), programmed death-ligand 1 (PD-L1), and cytotoxic T-lymphocyte-associated protein 4 (CTLA-4) (Aghamajidi and Vareki, 2022; Xie et al., 2025). This point is especially relevant to neuro-oncology because the systemic state of the immune system shapes the immune landscape of the CNS and immune cells’ capacity to respond to malignant cells.

Immunosuppression at the level of tumors may further enhance this connection. The immune response can be blocked due to the lack of interferon signaling and antigen presentation. Simultaneously, T regulatory cells and myeloid-derived suppressor cells are capable of recruiting and producing immunosuppressive agents, such as TGF-β, IL-10, and IDO. This way, a favorable immune environment for the development of the tumor is formed (Vinay et al., 2015). As a result, the microbiome-mediated modulation of the systemic immune response is connected with the existing immunosuppressive processes in the tumor microenvironment. Barrier regulation is another potentially significant connection. Among the microbiome-derived metabolites, SCFAs, namely acetate, propionate, and butyrate, have been associated with maintenance of BBB integrity and the regulation of communication between the gastrointestinal tract and the nervous system under both physiological and pathological conditions (Fock and Parnova, 2023). Because barrier disruption alters the trafficking of immune and soluble factors between the circulation and the CNS, changes in microbiome-derived metabolites could influence the inflammatory environment around brain tumors. However, the extent of the involvement of these mechanisms in human brain tumors needs to be clarified.

In relation to the tumor microenvironment of the brain, this systemic effect is linked to the highly specialized cells that exist in the microenvironment. Astrocytes and microglia play a role in the formation of the tumor microenvironment when gliomas develop, while TAMs constitute one of the most abundant populations of cells that are not neoplastic in GBM. This population of cells generates an environment that is characterized by inflammation and immunosuppression and drives tumor growth and resistance to treatment through cytokines and signaling pathways that promote tumors (Nóbrega et al., 2024; Ganesh et al., 2025).

### 2.3 Mitochondria as the mechanistic bridge between the microbiome and brain tumors

In this regard, the proposed link between microbial signals and brain-tumor biology becomes even more interesting, given that mitochondria are regarded as intracellular sensors and executors of metabolic and environmental information. Beyond ATP production via oxidative phosphorylation, mitochondria regulate metabolic intermediate availability, calcium homeostasis, ROS production, innate immunity, and apoptosis (Harrington et al., 2023). These roles imply the key role of mitochondria in metabolism, stress, and immune system regulation, the very functions which are known to be affected by the microbiome.

The communication of the microbiome with mitochondria happens in the intestinal epithelium, which makes it possible. As a result of the interconnection of epithelial hypoxia and microbiota in terms of metabolism, obligate anaerobes proliferate and produce SCFAs as their microbial metabolites. On the other hand, the signals coming from the microbiome can impact the mitochondrial ultrastructure and metabolic features, modify inflammasome activation, and change epithelial hypoxia (Zhang et al., 2022). Thus, this information shows that mitochondria can be used as cellular sensors to decode microbiome signals and trigger metabolic and inflammatory reactions (Jackson and Theiss, 2019).

In addition, such interconnection can happen in the other tissues and organs. Microbial metabolites can influence mitochondrial metabolism in the central nervous system. Therefore, the communication between the microbiome and mitochondria provides the mechanism to influence brain cellular physiology because of changes in the gut microbiome (Qiao et al., 2024). This interconnection is reciprocal since microbial metabolites affect the redox homeostasis and increase the mitochondrial ROS production, which is responsible for the cellular dysfunction and results in the development of the neurological diseases (Shandilya et al., 2021). On the other hand, there are some investigations that demonstrate the way how microbial metabolites reduce mitochondrial anomalies in the neurological diseases (Moretti et al., 2011). The research related to neurodegenerative diseases has shown the interrelation between gut microbial metabolites, cellular metabolism, and mitochondrial function in the CNS (Pereira et al., 2022).

Furthermore, certain microbial metabolites can connect the interrelation with the tumor and immune biology. For instance, butyrate regulates the systemic immune response, neuroinflammation, and stimulates Treg differentiation and their functions (Mafe and Büsselberg, 2025). In addition, microbes influence tryptophan metabolism and its interconnection with kynurenine pathway, which impacts immune suppression and tumor biology (Gupta et al., 2023). Therefore, based on the examples provided above, it can be stated that besides being metabolic substrates, the microbial metabolites can become signaling molecules that influence immune, inflammatory, and metabolic states related to tumor biology.

It is significant that the above evidence does not have equal levels of biological proof and cannot be compared directly to the evidence in GBM. Evidence in intestinal epithelium models shows the effect of microbial cues on mitochondrial physiology and inflammation, while evidence from neurological and neurodegeneration models has shown the effects of microbiota-derived metabolites on mitochondrial function and redox balance in the brain. All the above evidence serves as an important means to understand the biology of the relationship between the microbiome and mitochondria; however, it alone does not provide sufficient evidence of how the two communicate in GBM cells or GBM-related immune cells (Ganesh et al., 2025).

This metabolic interface is especially important in glioblastoma (GBM). Indeed, GBM cells require metabolic reprogramming for proliferation, invasion, biosynthesis, and adaptive responses to therapy-induced stress (Morrow et al., 2021). Changes in glycolysis, oxidative phosphorylation, PPP, fatty acid synthesis and oxidation, and nucleotide metabolism provide GBM with metabolic plasticity (Morrow et al., 2021). Although the Warburg effect is often described as aerobic glycolysis being a hallmark of cancer metabolism, recent findings show that some tumor populations can rely heavily on oxidative phosphorylation, indicating that glycolysis and mitochondrial metabolism can coexist and dynamically shape tumor bioenergetics. In this case, the metabolic flexibility influences the mitochondria. Therefore, the changes associated with mitochondria in GBM and other cancers involve the dependence on OXPHOS, the production of reactive oxygen species, mitochondrial morphology, mtDNA stability, apoptosis, and the relationship between glycolysis and oxidative ATP synthesis (De Oliveira et al., 2024). As seen, the mitochondria are not only the organelles responsible for energy production but also they may affect metabolic plasticity and survival of cancer cells. In this respect, microbial metabolites and inflammation may be considered as upstream modulators of the state of mitochondria (**Figure 1**).

**Figure 1:**
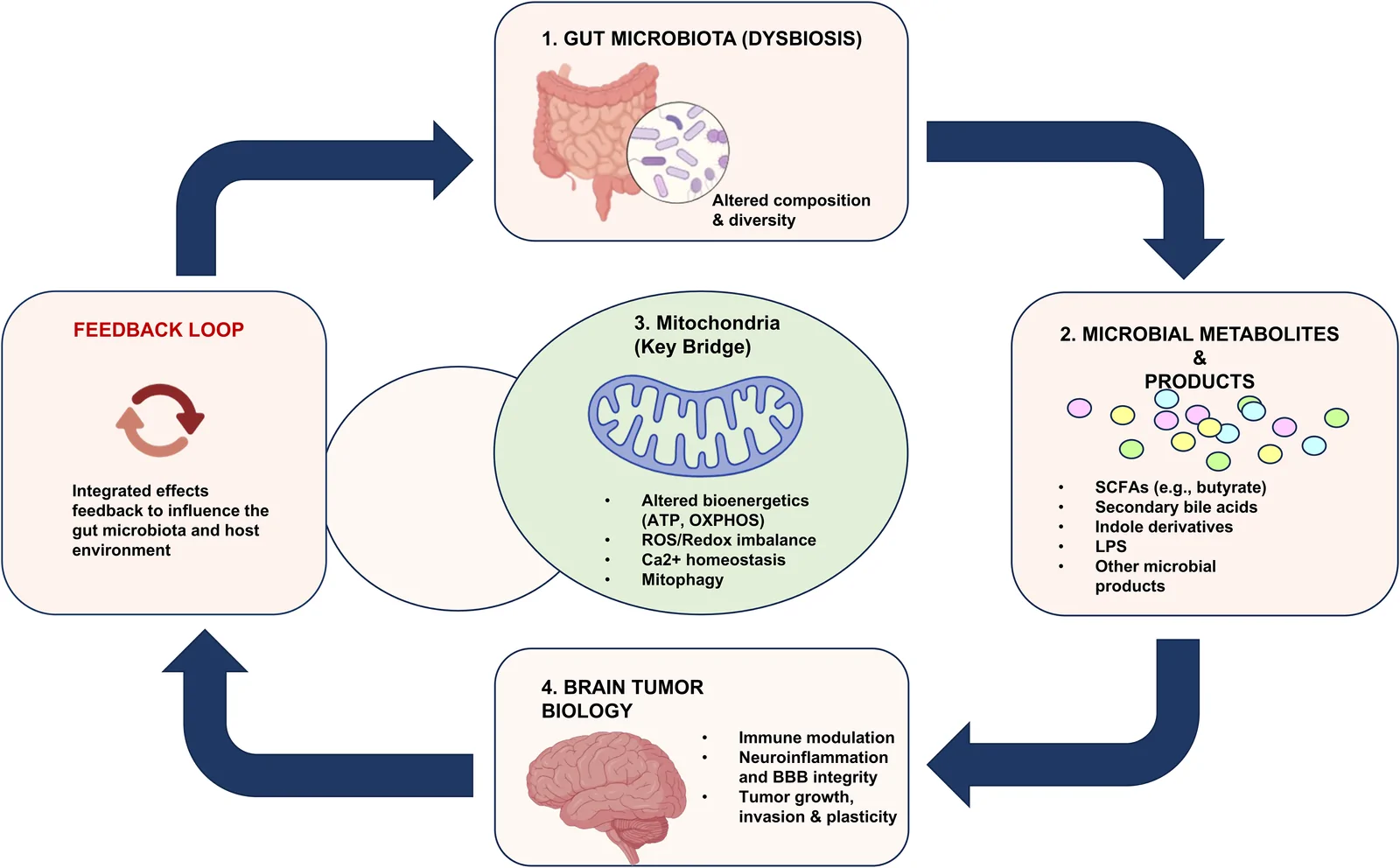
Schematic representation of microbiome-mitochondria crosstalk in brain tumor biology. Gut microbial metabolites influence mitochondrial homeostasis, which modulates neuroinflammation, immune responses, and tumor progression through a feedback loop.

Together, these facts suggest that the gut microbiome can influence neuro-oncology through metabolic, immune, barrier, and mitochondrial pathways, with mitochondria as a point of mechanistic convergence between systemic microbial signals and cellular programs in brain tumors. However, direct evidence linking microbiome changes to mitochondrial phenotypes in brain tumors remains limited. Therefore, the next stage would be to investigate the molecular mechanisms by which microbiome-derived signals interact with mitochondrial metabolism, dynamics, redox signaling, and cellular stress signaling, and how these contribute to brain tumor biology.

## 3 Molecular mechanisms underlying microbiome-mitochondria crosstalk

Communication between gut microbiota and host mitochondria has long been recognized to be bidirectional rather than unidirectional, owing to metabolic by-products of microflora impacting the host cells. It has been shown that the metabolites produced by gut microbiota could influence the activity of mitochondria in neurons and glia, whereas mitochondrial malfunction would impact the epithelial and microbial milieu through secretion of damage-associated molecular patterns and other stress mediators (Qiao et al., 2024; Kulkarni and Gaikwad, 2025). Such a form of communication could be a way how gut microbiota could impact metabolism, redox signaling, inflammation, and cell fate. This is important for neuro-oncology, since tumor metabolism and signaling, along with the condition of nearby immune and glial cells, determine tumor development.

It is worth noting that the evidence regarding interactions between the microbiome and mitochondria is obtained through several biological systems, and all of them should not be regarded as equal in strength. In this review, GBM-specific evidence is defined as the evidence which was obtained from human GBM cohorts, GBM-specific material obtained from patients, glioma-specific cellular and animal models, and microbiome-associated studies related to gliomas. In turn, results from other cancers, neurological diseases, intestinal model systems, immune cell-based systems, and other non-GBM experimental models should be viewed as complementary evidence rather than direct proof of the interaction between the microbiome and mitochondria in GBM.

### 3.1 Microbial metabolites and mitochondrial bioenergetics

In terms of the different modes through which the gut microbiota interacts with the host, the microbial metabolites can be considered as one of the most crucial ones (Lavelle and Sokol, 2020; Swer et al., 2022). Based on the particular metabolite and its systemic and local concentrations, microbial metabolites have the ability to impact the host’s metabolism, immune development, mucosal barrier, and inflammatory signaling (Lavelle and Sokol, 2020; Swer et al., 2022). In particular, SCFAs and secondary bile acids should be highlighted among microbial compounds since they can influence metabolism and mitochondrial processes in neurons and glia (Qiao et al., 2024).

Through substrate availability and signaling pathways, microbial metabolites can affect mitochondrial bioenergetics in multiple ways, including changes in mitochondrial respiration, oxidative stress, fission-fusion balance, epithelial integrity, immune response, and inflammatory pathways (Yosefi et al., 2026). On the contrary, the metabolic activity of the microbiome is strongly correlated with mitochondrial metabolism of the host (Weber-Stiehl et al., 2022), thus forming the interdependence between the microbiome and host metabolism when microbial communities supply the metabolic substrates and signaling molecules capable of changing host mitochondrial function (Weber-Stiehl et al., 2022).

A clear example is the action of SCFAs. Acetate, propionate, and butyrate can be formed as a result of microbial fermentation of host substrates (Lavelle and Sokol, 2020; Swer et al., 2022). Butyrate is especially important for colon function because it is an important substrate for colonic epithelial cells (Besten et al., 2013). Butyrate can also influence cellular metabolism and mitochondrial function. Experimental data show that butyrate can inhibit hexokinase 2 activity by interacting with HDAC8, thereby affecting cell survival and respiration during inflammation (Hinrichsen et al., 2021). This example shows how microbial metabolites can affect not only cellular metabolism but also epigenetic regulation and mitochondrial respiration under cellular stress.

Bile acids are another example of the pathway through which microbial metabolism affects mitochondrial function. Primary bile acids, namely cholic and chenodeoxycholic acids, are synthesized by the hepatocytes of the host from cholesterol and then transformed into secondary bile acids (deoxycholic acid [DCA] and lithocholic acid) by intestinal microbial communities (Winston and Theriot, 2019). Because secondary bile acids can interact with nuclear and membrane receptors, changes in bacterial bile acid metabolism may affect systemic metabolic and immune signaling (Fiorucci et al., 2026). Furthermore, secondary bile acids could influence the functioning of mitochondria by modulating mitochondrial biogenesis, oxidative phosphorylation, mitochondrial membrane potential, and mitochondrial mass; decreasing respiration; and increasing the formation of mitochondrial reactive oxygen species (Abrigo et al., 2023). Modulation in microbial-dependent bile acid profile could thus be one mechanism through which dysbiosis influences mitochondrial metabolism and reactive oxygen species formation.

The issue becomes even more interesting when discussing neuro-oncology due to the fact that mitochondrial metabolism controls ATP production, reducing power, biosynthetic substrates, and ROS signaling pathways, thus controlling the capacity of tumor cells to adapt. However, there is different amounts of data based on the type of model being used. Even though the impact of microbial metabolites on the processes of mitochondrial respiration, redox state, and related metabolic pathways has already been confirmed, most of the mechanistic insights were gained with the use of intestinal, immune, neurological, or not GBM models. Such findings are certainly interesting from the biological perspective but cannot be regarded as evidence of the presence of similar mechanisms in human GBM cells.

### 3.2 Regulation of mitochondrial dynamics, biogenesis, and mitophagy

Apart from respiration, mitochondria are controlled by frequent mitochondrial dynamics that involve fission and fusion, responsible for maintaining the quality of mitochondria, the distribution of mitochondrial constituents, and stress adaptation of cells. Among the regulators of fission are DRP1, whereas MFN1/MFN2 regulate the outer membrane fusion, and OPA1 is involved in inner membrane fusion (Van Der Bliek et al., 2013). Disruption of the regulation of these events has been observed in cancer and neurological disorders (Adebayo et al., 2021).

Gut microbiota metabolites may affect mitochondrial remodeling in the same way as host-produced metabolites by directly modifying the mitochondrial network. For example, butyrate has been shown to increase mitochondrial fission and alter mitochondrial ultrastructure and function in human monocytes (Pérez-Hernández et al., 2022). Although it is impossible to state a similar effect in brain tumors, microbiome-derived metabolites can directly influence mitochondrial structure and function in immune cells. This is especially important in the tumor microenvironment, where mitochondrial remodeling controls immune cell function.

Regulation of mitochondrial capacity through mitochondrial biogenesis is another strategy that could be used. The signaling pathways involved in mitochondrial and nuclear regulation of biogenesis involve ATP signaling, ROS signaling, changes in Ca^2+^ levels, and transcription factor regulation of mitochondrial biogenesis (Zhao et al., 2023b). Membrane potential changes in the mitochondria could affect the ability of the mitochondria to absorb Ca^2+^ and thus activate Ca^2+^ dependent pathways to regulate biogenesis (Liu et al., 2023). There is an important regulatory pathway involving the PGC-1 that is involved in the stimulation of the expression and activity of nuclear respiratory factors and hence, TFAM-dependent regulation of mtDNA replication and transcription (Wu et al., 1999). In this case, disruption of these pathways would reduce the mitochondria’s capacity to adapt to metabolic stress and oxidative metabolism.

Another aspect of mitochondrial function maintenance is selective autophagy, or mitophagy, which identifies and removes damaged mitochondria. It is essential for maintaining mitochondrial OXPHOS, ATP production, Ca^2+^ homeostasis, ROS control, and mtDNA integrity (Picca et al., 2023). Accumulation of the PINK1 protein on the surface of damaged mitochondria activates Parkin-dependent autophagy of the organelles (Rüb et al., 2016). Any dysregulation of this process would affect the mitochondrial state in cells. The impact that microbiome metabolites may have on mitochondrial quality control might occur indirectly due to alterations in the membrane potential of mitochondria, the production of reactive oxygen species, cellular energy levels, and inflammation signaling. Such changes might have an impact on the recognition and elimination of defective mitochondria through mitophagy, involving the PINK1-Parkin pathway, for example. Mitophagy alterations would then influence mitochondrial mass, ROS balance, and metabolism. Nevertheless, there is no clear information about the impact of microbiome metabolites on mitophagy in GBM.

Overall, the effects of microbe-derived metabolites and systemic microbiota-derived signaling molecules could impact mitochondrial activity through pathways that interconnect bioenergetics, redox signaling, mitochondrial dynamics, biogenesis, and mitophagy. Impacts on these pathways could potentially affect mitochondrial integrity, ROS homeostasis, and metabolic flexibility. However, there is currently limited experimental evidence supporting a complete microbiome-mitochondria connection in the context of metabolic reprogramming in GBM (**Figure 2**).

**Figure 2:**
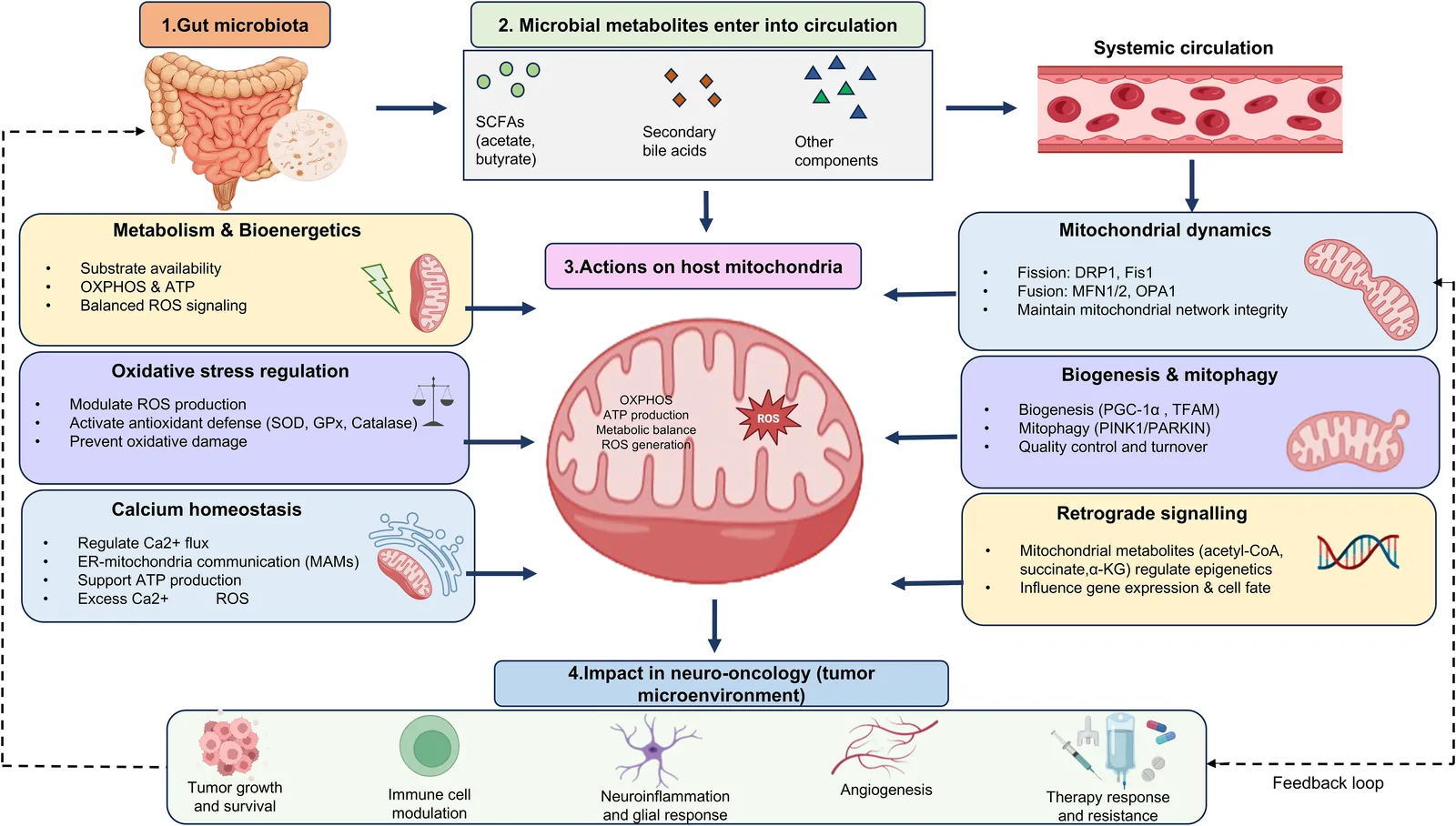
Schematic illustration of microbial metabolite-mediated regulation of host mitochondrial function in neuro-oncology. Gut microbiota-derived metabolites enter systemic circulation and modulate mitochondrial metabolism, redox balance, calcium homeostasis, dynamics, biogenesis, mitophagy, and retrograde signaling. These mitochondrial changes influence the tumor microenvironment, promoting immune modulation, neuroinflammation, angiogenesis, tumor progression, and therapeutic response.

### 3.3 Oxidative stress, redox homeostasis, and mitochondrial signaling

Oxidative phosphorylation of mitochondria is directly connected with redox signaling. Mitochondrial respiration produces not only toxic products, such as reactive oxygen species, but also signaling compounds in a controlled manner when their production is moderate, which contributes to adaptation processes caused by changes in metabolism and environment (Palma et al., 2023). On the other hand, ROS formation in an uncontrolled manner can cause imbalance in cellular functions and oxidative stress (Palma et al., 2023; Sena and Chandel, 2012). Also, changes in microbiota composition can affect redox balance in mitochondria and vice versa (Ballard and Towarnicki, 2020). There exist several ways to regulate the cell redox balance. In particular, there are antioxidants that allow decreasing superoxide radicals to hydrogen peroxide by means of superoxide dismutases and further reducing hydrogen peroxide with catalase and glutathione peroxidases (Sies, 1997; Papas et al., 2019). Besides, the aforementioned systems do not only reduce ROS but also regulate the level of ROS in a way that prevents oxidative stress and allows signaling.

Microbiome-associated signals can be linked with the redox network described above with the help of some molecular mediators. First, there are microbiome signals that influence ROS generation by mitochondria and redox-active molecules, including nitric oxide, hydrogen sulfide, and short-chain fatty acids, that also take part in microbiome-mitochondria signaling (P. Gupta et al., 2025). Disruption of such a system can cause inflammation, oxidative stress, and mitochondrial dysfunctions. At the same time, antioxidants, including SOD, GPx, and catalase, are tightly regulating mitochondrial ROS.

The redox interface is especially pertinent to tumor biology since tumor cells thrive in a state of metabolic stress due to varying oxygen supply, starvation and oxidative stress and thus need great deal of redox plasticity. Metabolites and signals generated from the microbiome, which modulate respiratory function or substrate metabolism in mitochondria, may potentially affect ROS generation and antioxidants in mitochondria. ROS generated by the mitochondria can serve both as markers of metabolic stress as well as mediators of stress response, inflammation, proliferation and survival. Within the brain-tumor microenvironment, redox signaling can further impact immune and glial cell physiology. However, contribution of the microbiome in metabolic reprogramming of ROS-dependent metabolism in GBM remains unclear.

### 3.4 Calcium signaling and mitochondria-associated membranes

Ca^2+^ is one of the important second messengers inside cells, enabling the coordination between extracellular and intracellular signaling and controlling metabolism, proliferation, stress reaction, and cell fate (Bootman and Bultynck, 2019). The transport of Ca^2+^ to mitochondria is an essential metabolic pathway connecting signaling in the cytoplasm with cellular respiration in mitochondria. A moderate increase in mitochondrial Ca^2+^ increases the activity of dehydrogenases and favors oxidation of metabolism, providing more reducing equivalents for ATP synthesis. But an overabundance of Ca^2+^ can increase ROS generation and induce mitochondrial dysfunction and cell death (Tarasov et al., 2012).

As one of the key intracellular Ca^2+^ stores, the endoplasmic reticulum is responsible for the transfer of Ca^2+^ and other signaling molecules through a complex of specialized areas of interaction between the endoplasmic reticulum and mitochondria called mitochondria-associated membranes (MAMs) (Groenendyk and Michalak, 2023; Patergnani et al., 2011). Ca^2+^ uptake into mitochondria occurs via a calcium uniporter (MCU) complex, comprising MCU and its regulators - MCUb, EMRE, MICU1, and MICU2- contributing to the regulation of mitochondrial Ca^2+^ uptake and adaptation to metabolic needs (Pathak and Trebak, 2018).

As seen from experimental data, microbial signals can influence this phenomenon. In this case, A. muciniphila and its conditioned media interfere with the Ca^2+^ balance inside the cell as well as increase the levels of Ca^2+^ in mitochondria of enteroendocrine cells. The increased level of Ca^2+^ is associated with oxidative stress and mitochondrial dysfunction (Neto et al., 2022). In neuro-oncology, this pathway may be important because Ca^2+^ signaling and mitochondrial metabolism are closely linked to tumor cell survival and stress responses. However, direct evidence that gut microbial signals can modify MAM-dependent Ca^2+^ signaling in brain tumor cells remains limited. Existing evidence only serves as a basis for investigating this hypothesis.

### 3.5 Mitochondrial retrograde signaling and nuclear reprogramming

In addition, mitochondria can signal to the nucleus via retrograde signaling pathways, which involve communication between mitochondria and the nucleus in which information about mitochondrial function is conveyed through regulation of gene expression. Disrupted mitochondrial function might result in disruptions in redox state, concentration of Ca^2+^, ATP-to-AMP ratio, as well as concentrations of metabolic intermediates in mitochondria (Hunt and Bateman, 2017; Walker and Moraes, 2022). These factors can affect the activity of transcriptional regulators, altering chromatin remodeling and gene transcription related to metabolism, inflammation, stress, and cell survival (Datta et al., 2022).

Metabolites produced by mitochondria serve as intermediaries in the communication between mitochondria and the nucleus. For example, fumarate, acetyl-CoA, succinate, and α-ketoglutarate can influence epigenetic enzyme activity and induce transcriptional changes driven by alterations in mitochondrial metabolism (Santos, 2020). Thus, persistent alterations in mitochondrial function lead to metabolic and epigenetic adaptations that affect cell phenotype and contribute to malignancy in certain pathological conditions. This pathway represents one possible way by which the microbiome-derived signals might mediate tumor-cell metabolic reprogramming. Since any alteration in the mitochondrial metabolism mediated by the microbial metabolites can have an impact on the mitochondrial metabolites and the energy states, these alterations will be able to influence the transcriptional and epigenetic regulation in the nucleus. As a result, mitochondrial-nuclear communication can have an impact on the cell’s glycolysis, oxidative phosphorylation, biosynthesis, stress signaling, inflammation signaling, and even survival mechanisms. However, there is no concrete experimental evidence for this entire microbiome-mitochondrial-metabolic reprogramming pathway in GBM.

### 3.6 Mitochondrial control of cell death and survival pathways

Mitochondria serve as a decision point for cell survival or programmed cell death. The intrinsic apoptotic pathway regulates the interaction of pro- and anti-apoptotic members of the BCL-2 protein family (Li and Dewson, 2015). With appropriate apoptotic stimuli, BAX and BAK cause MOMP, releasing intermembrane proteins such as cytochrome c, SMAC, and OMI. Cytoplasmic cytochrome c binds APAF1 protein and activates formation of the apoptosome and initiation of caspase-9, which further activates executioner caspases such as caspase-3 and caspase-7 (Vringer & Tait, 2023).

Therefore, mitochondria act as a target where various metabolic, oxidative, and stress signals converge to decide the cell fate. Mitochondrial structural and functional plasticity allows cells to respond to signals and either maintain metabolic adaptation or progress to cell death (Sedlackova and Korolchuk, 2018). Beyond apoptosis, mitochondria also participate in stress responses and help execute different types of cell death and autophagy to maintain tissue homeostasis (Galluzzi et al., 2012). Thus, in microbiome-mitochondria communication, microbial metabolites and signals might affect this survival-decay balance by regulating mitochondrial metabolism, oxidative stress, and apoptosis signaling. Such changes may be especially important in tumors, where resistance to mitochondrial stress and apoptosis plays a crucial role in therapy resistance (Galluzzi et al., 2012). In turn, dysfunction of mitochondria and generation of danger signals might affect the inflammatory and barrier responses and thus change the interactions of the host and the microbiome (Jackson and Theiss, 2019). Whether these reciprocal mechanisms contribute to the survival and therapeutic resistance of brain-tumor cells remains unknown. Taken together, the available evidence indicates that microbial metabolites and microbial-derived signals can influence mitochondrial bioenergetics, redox homeostasis, calcium signaling, mitochondrial dynamics, biogenesis, and quality control across several experimental systems. However, these mechanisms are supported predominantly by intestinal, immune, neurological, and other non-GBM models, whereas direct evidence in human GBM remains limited. **Table 1** summarizes these mechanistic relationships and explicitly distinguishes the experimental systems from their potential relevance to GBM (**Table 1**).

**Table 1:** Microbial metabolites and microbial-derived signals, mitochondrial targets, experimental models, and potential relevance to GBM.

Microbial metabolite/signal	Mitochondrial target or process	Experimental model/system	Observed mitochondrial effect	Potential relevance to GBM	Evidence level	Supporting reference
SCFAs, particularly butyrate	HK2-HDAC8 axis; glycolytic- mitochondrial coupling; mitochondrial respiration	Caco-2 cells, intestinal epithelial organoids, Hk2ΔIEC mice	Butyrate represses HK2 through HDAC8 and reduces mitochondrial respiration; effects are linked to epithelial metabolic adaptation	Provides a mechanistic example of how a microbiota-derived metabolite can alter cellular glycolysis-mitochondrial coupling; no direct GBM evidence	Non-GBM experimenta l evidence	Hinrichsen et al., 2021
Butyrate	Mitochondrial dynamics and ultrastructure	Human monocytes	Increased mitochondrial fission and altered mitochondrial ultrastructure and function	May be relevant to mitochondrial remodeling of tumor-associated immune cells; direct effects in GBM remain unestablished	Immune-cell evidence	Pérez-Hernández et al., 2022
Bile acids, particularly DCA and CA	Mitochondrial membrane potential, OXPHOS, mitochondrial mass, biogenesis, mtROS, mitophagy/auto phagic flux	C2C12 myotubes; mouse model of cholestasis-associated sarcopenia	Reduced mitochondrial mass, membrane potential and respiration; reduced OXPHOS complexes and biogenesis; increased mtROS and altered mitophagy/aut ophagic flux	Demonstrates that bile-acid signaling can coordinately affect mitochondrial bioenergetics, ROS and quality control; not demonstrated in GBM	Non-neural experimenta l evidence	Abrigo et al., 2023
Microbiome-derived redox-active signals (SCFAs, H_2_S and related microbial signals)	Mitochondrial ROS generation and redox homeostasis; antioxidant responses	Host-microbiome and cellular experimental systems	Microbiome-associated signals can influence ROS/redox balance and mitochondrial stress responses	Provides a mechanistic basis for potential effects on GBM redox adaptation and tumor-immune interactions, but direct GBM validation is lacking	Complemen tary mechanistic evidence	Gupta et al., 2025
Akkermansia muciniphila-derived microbial products	Mitochondrial Ca^2+^ homeostasis and Ca^2+^-dependent mitochondrial stress	STC-1 enteroendocrin e cells; mouse model	Conditioned medium increased mitochondrial Ca^2+^ uptake, mitochondrial stress and ROS generation, with reduced mitochondrial membrane potential	Supports a possible link between microbial products, Ca^2+^ signaling and mitochondrial dysfunction; effect has not been demonstrated in GBM cells	Intestinal/ne urodegenera tive mechanistic evidence	Neto et al., 2022
Gut microbial metabolites influencing CNS redox metabolism	Mitochondrial redox homeostasis and ROS regulation	CNS/neurolog ical and neurodegenera tive experimental systems	Microbial metabolites can influence mitochondrial metabolism and oxidative stress in the CNS	Provides mechanistic plausibility for microbiome-mitochondria communication in neural tissues; does not establish the pathway in GBM	Neurologica l evidence	Qiao et al., 2024; Shandilya et al., 2021; Pereira et al., 2022
Tryptophan-derived microbial metabolites	Host metabolic and inflammatory signaling; potential interaction with mitochondrial/r edox pathways	Microbiome- immune/neuro biological systems	Microbial transformation of tryptophan influences kynurenine- related and immune-metabolic pathways	May influence GBM-associated immunosuppression and metabolic adaptation; direct microbiome-mitochondria effects remain insufficiently defined	Indirect/co mplementar y evidence	Gupta et al., 2023; Mafe and Büsselberg, 2025

## 4 Microbiome-mitochondria crosstalk in brain tumor progression

However, microbiome-mitochondria interactions in neuro-oncology are important for understanding mitochondrial adaptation within the metabolic and functional plasticity of brain tumors. Glioma cells constantly adapt their bioenergetic and biosynthetic pathways in response to nutritional, oxygen, and inflammatory factors, as well as therapeutic stress. Because microbiome-derived metabolites affect host metabolism, redox state, and mitochondrial function, they may serve as additional signals from the systemic environment that influence adaptation. However, the strength of evidence supporting such proposed links is not consistent across biological systems. Specific studies in GBM show evidence for mitochondrial metabolic flexibility and links between the microbiome, tumors, and drug responses, while mechanistic studies of specific links between microbiome signals and mitochondrial programming in GBM have yet to be performed. Mechanistic evidence from other cancers, neurological diseases, *in vitro* intestinal models, and non-GBM experimental models contributes to biological plausibility of this link, but should not be used as direct evidence of microbiome-mitochondria interactions in GBM. Therefore, the effects of interactions between the microbiome and mitochondria on tumor metabolism, proliferation, invasion, stem cells, hypoxia adaptation, and drug resistance should be viewed as a promising framework requiring GBM-specific validation (Dubiński et al., 2025; Ganesh et al., 2025).

### 4.1 Metabolic reprogramming in brain tumors

Metabolic rewiring is another hallmark that shapes glioma biology by providing energy and biomolecular substrates to cancer cells in response to proliferative processes within a constantly changing environment. The energy production, metabolism substrate, and reductants generated by aerobic glycolysis (Warburg effect) in combination with the redirection of glycolysis intermediate metabolites to other pathways (pentose phosphate pathway), which allows generating nucleotides and maintaining redox equilibrium (Venneti and Thompson, 2017), and glioma cells’ growth is ensured by using glucose, amino acids (glutamine, serine, glycine, cysteine), and aberrant lipid metabolism (Lu et al., 2024). These pathways are linked through the tricarboxylic acid cycle that furnishes carbon skeletons and reducing power (Trejo-Solís et al., 2023, 2024). In turn, the microbiome meets tumor cells’ metabolic needs in two ways: systemic metabolism and reprogramming of immune cells. First, gut dysbiosis leads to lower SCFA concentrations in blood and tumors, as well as an increased number of M2-like macrophages in the TME. iSCFAs affect tumor-associated macrophage metabolism by increasing glycolytic activity M1 promoting polarization (Zhou et al., 2024). Thus, this case illustrates how microbial metabolites affect tumor cell metabolism by altering immune-cell metabolic states rather than tumor-cell metabolism. This raises an interesting question about how microbiome metabolites act in glioma cells. They can act at two interconnected levels: directly altering tumor cell metabolism and reprogramming immune and stromal cell metabolism in the TME. However, evidence for direct reprogramming of mitochondrial metabolism by specific microbial metabolites in human glioma cells remains insufficient.

### 4.2 Tumor proliferation, invasion, and cellular plasticity

The formation of brain tumors is linked to metabolic processes not only in terms of biomass creation but also in terms of adaptation to the tumor environment. Despite the enhanced glycolytic pathway in gliomas, mitochondrial oxidative metabolism is vital for the development of tumors (Venneti and Thompson, 2017). In addition to synthesizing ATP, mitochondria provide metabolic precursors for lipids, amino acids, nucleotides, and other cellular components (Ahn and Metallo, 2015). For instance, in GBM, mitochondria provide coenzyme Q for the redox cycle and support the respiration-dependent activity of dihydroorotate dehydrogenase to ensure pyrimidine *de novo* synthesis. Therefore, mitochondria can contribute to the biosynthesis of compounds required for tumor development. Impaired mitochondrial respiration negatively affects the tumor-forming ability of GBM cells (Luo et al., 2026). Mitochondrial activity can also affect tumor cell signaling by altering ATP levels and ROS production. Signals produced by mitochondria affect pathways of proliferation, survival, and adaptation to environmental changes (in terms of nutrients and oxygen availability) that enable tumor cells to keep growing under heterogeneous microenvironmental conditions (Luo et al., 2026). This is important in GBM because GBM cells can adapt to environmental heterogeneity during migration.

Tumor infiltration into the neighboring brain tissue is one of the characteristic features of GBM that makes tumor boundaries undefined and results in the impossibility of total resection (Seker-Polat et al., 2022). ECM composition, stiffness, porosity, and topography can affect GBM cell migration through surrounding tissue (Ulrich et al., 2009). Mitochondrial metabolic adaptability and changes in their dynamics may also support the energetics and signaling processes linked to tumor cell migration and invasion (Ulrich et al., 2009; Seker-Polat et al., 2022). This plasticity is not limited to just invasion. GBM cells can reversibly modulate their metabolism and phenotypic state in response to varying microenvironmental factors, enabling different cellular populations to cope with oxygen, nutrient, and therapy stress. This plasticity enables GBM to progress, evade therapy, and recur. It may involve coordinated energy production and synthesis of biochemical compounds (Yabo et al., 2022). Within the hypothesized microbiome-mitochondria axis, microbial metabolites may further influence this adaptability. However, this relation is mechanistically possible but not yet established in human GBM.

### 4.3 Cancer stem cells and tumor heterogeneity

GBM heterogeneity is strongly associated with the presence of self-renewing, pluripotent, propagating, and chemoresistant/radiation-resistant glioblastoma stem cells (GSCs) in the tumor (Jin et al., 2021). These cells play an important role in tumor development and its recurrence. GSCs do not share a common metabolic profile; they use mitochondria to maintain stemness and protect against stress, while their cell state depends on either glycolysis or oxidative metabolism (Iranmanesh et al., 2021). Mitochondrial redox regulation and stress response pathways are vital for the survival of GSC populations under hypoxic and radiative conditions. Thus, GSCs’ ability to maintain their mitochondrial state and adapt energy-producing pathways under environmental stress contributes to the persistence of tumor-propagating cell populations. GSC populations can switch their bioenergetics and redox metabolism, providing additional evidence for a link between mitochondrial state and cellular identity.

Heterogeneity of GBM is significant to note here. Genomic instability, clonal evolution, and microenvironmental interactions lead to heterogeneous cells in the tumor (Zhang et al., 2026). There can be variations in mitochondrial and metabolic characteristics of cells that allow them to alter their bioenergetics and redox metabolism based on the local microenvironment. Thus, response to microbe signaling is not universal and depends on the particular metabolic/differentiation status of the cell. This factor is critical to the microbiome-mitochondria hypothesis because it suggests that microbial signals may not affect the whole tumor’s metabolism but instead drive metabolic selection. Further research should determine whether particular microbial metabolites preferentially support GSCs, mitochondrial plasticity, or therapy-resistant states (**Figure 3**).

**Figure 3:**
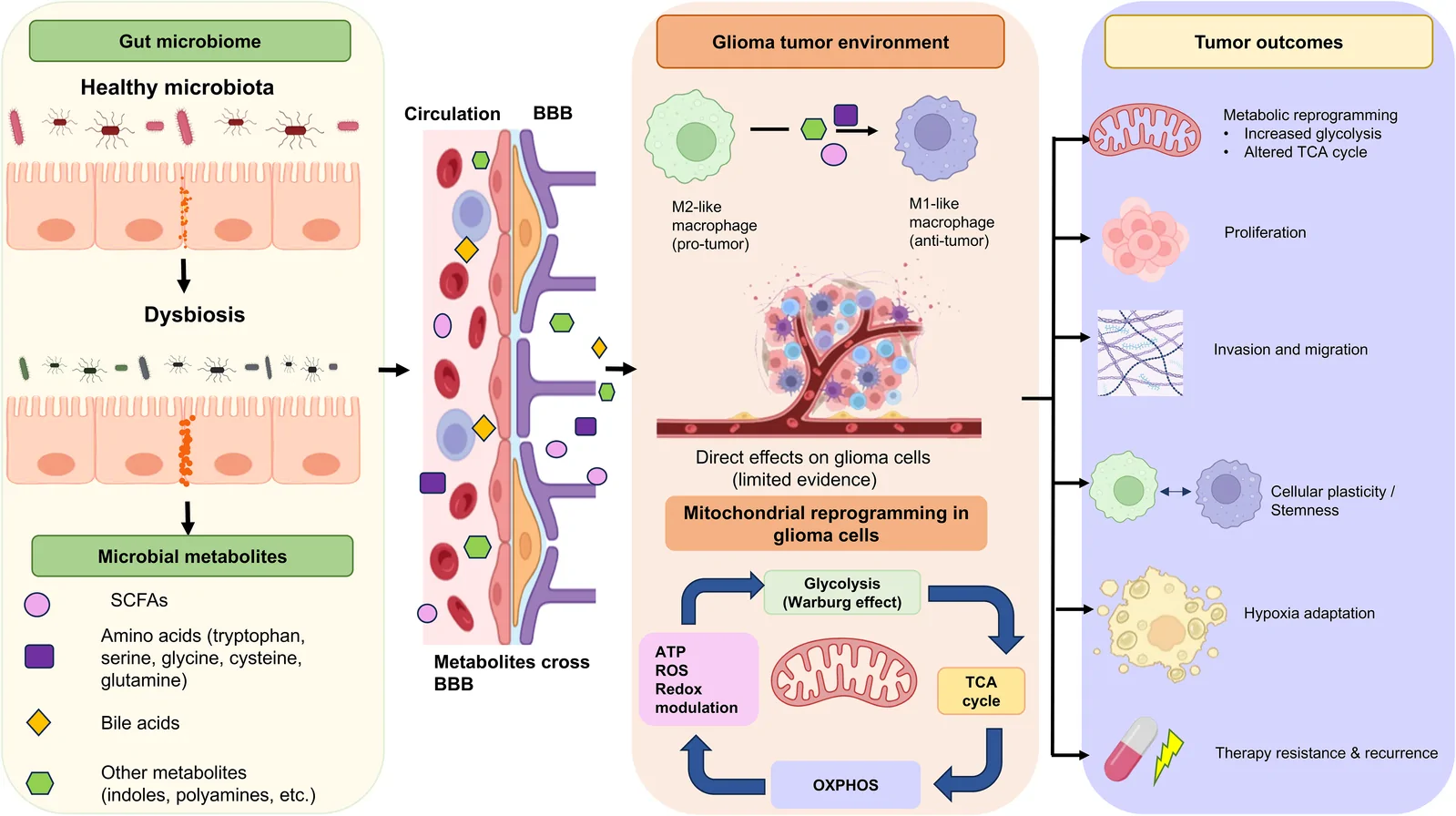
Schematic overview of gut microbiome-mediated mitochondrial reprogramming in glioma. Microbial metabolites generated during dysbiosis cross the blood-brain barrier and reshape mitochondrial metabolism and immune signaling within the glioma microenvironment. These changes promote metabolic reprogramming, proliferation, invasion, hypoxia adaptation, cellular plasticity, and resistance to therapy.

Since the persistence of GSCs is strongly correlated with the risk of tumor recurrence, microbial regulation of mitochondrial plasticity may be a key element that connects microbe-derived signals, stemness properties, and recurrence. For instance, microbial metabolites might modulate substrate utilization, OXPHOS efficiency, ROS production, and mitochondria-related degradation pathways in metabolically flexible glioma cells (Qiao et al., 2024; Koynova-Tenchov, 2026). Changes in these metabolic properties might alter the glycolytic versus oxidative phenotype balance in GSCs and, consequently, the ability of these stem cell populations to preserve stem properties and survive hypoxic, radiation-induced, or chemotherapy-induced stress. The metabolic plasticity of GSCs, including the capability of these cells to switch to glycolysis upon hypoxia or OXPHOS inhibition, serves as a basis for this type of adaptation (Storevik et al., 2026). Increased mitochondrial reserve was shown to be responsible for radioresistance in experimental GSC models (Vlashi et al., 2011). As a result, a population of tumor cells which possesses the capacity for metabolic plasticity and the preservation of mitochondrial function during transitions between glycolytic and oxidative phenotypes may possess the capacity for persistence and recurrence of the tumor growth. However, there is currently no direct evidence that the specific metabolites of the gut microbiome regulate the maintenance or recurrence of GSCs due to mitochondrial reprogramming in GBM. Hence, this putative connection between microbiome, mitochondria, GSCs, and tumor recurrence should be considered as a new hypothesis (Ganesh et al., 2025).

### 4.4 Hypoxia, angiogenesis, and metabolic adaptation

The presence of hypoxia is a hallmark of GBM pathology. Even though there is extensive vascularization, irregular perfusion leads to the uneven availability of oxygen and nutrients, thus resulting in metabolic adaptation and cell death (Domènech et al., 2021). The mechanisms of hypoxia response that include regulation of metabolism, stemness, invasion, angiogenesis, autophagy, and immunosuppression allow for cell survival in the case of GBM (Bou-Gharios et al., 2024). The hypoxia-driven metabolic adaptation of tumors results in an increase in the proportion of glycolysis and lactate production. Lactate transport via monocarboxylate transporters MCT1 and MCT4 depends on the cell type and oxygen availability (Miranda-Gonçalves et al., 2016). Consequently, the level of oxygen affects the ratio of glycolysis and mitochondria-based metabolism.

The hypoxia-induced activation of HIF-1α signaling allows for the promotion of GBM angiogenesis via the secretion of proangiogenic factors like PDGF and VEGF. Angiogenesis in GBM includes three pathways: VEGF-VEGFR, angiopoietin-Tie, and Delta-Notch (Clara et al., 2014; Ramakrishnan et al., 2014; La Mendola et al., 2022). In addition, there is evidence suggesting that the process of mitochondrial quality control occurs under hypoxic conditions. For instance, NIX-dependent mitophagy helps get rid of damaged mitochondria in hypoxic and oxidative stress environments and allows the preservation of stem-cell population in cancers (Jung et al., 2019). These metabolic adaptations allow for the development of metabolic plasticity, invasion, stemness, and radio- and antiangiogenesis resistance (Mahase et al., 2017).

### 4.5 Therapeutic resistance and disease recurrence

Treatment of GBM currently includes maximal surgical removal of the tumor, radiotherapy, and temozolomide (TMZ) with Tumor-Treating Fields in some clinical settings. Still, treatment resistance and disease recurrence are the main difficulties. The blood-brain barrier (BBB), tumor heterogeneity, glioma stem cells, and tumor microenvironment (TME) interactions play the role in treatment resistance (Sherman et al., 2024). Treatment resistance is often observed in GBM patients even after multimodality therapy, and recurrence is challenging to treat (Davis, 2016). There are a number of reasons behind TMZ resistance. They include methylation by O6-methylguanine-DNA methyltransferase, DNA repair failure, transcriptional responses to hypoxia, and activation of different pathways like NF-κB, Hippo, and Wnt signaling pathway (Durga and Pusapati, 2025). Mitochondrial pathways constitute an additional feature of treatment adaptation. Changes in mitochondria-related ROS generation, metabolic switch, apoptosis, mitochondrial biogenesis and dynamics, mitochondrial stress response, and mtDNA were found to play a role in TMZ treatment resistance (H. Li et al., 2023; Chien et al., 2022). In general, metabolic plasticity enables TMZ-resistant cells to switch between metabolic pathways due to environmental and treatment-induced stress (Zampieri et al., 2021).

The microbiome might serve as another level in this adaptive network. The metabolic products of microorganisms, such as SCFAs, secondary bile acids, and tryptophan derivatives, can have an effect on host metabolic pathways and thus impact cellular responses to metabolic and therapeutic stresses (Koynova-Tenchov, 2026). In the case of GBM, they can potentially influence mitochondrial respiration, ROS balance, metabolic plasticity, apoptosis, and mitochondrial quality control - the processes that play a role in TMZ resistance. It is important due to the fact that the TMZ-resistant GBM cells display metabolic reprogramming in glycolysis, TCA cycle, fatty-acid metabolism, and amino-acid metabolism (Tomar et al., 2025), and mitochondrial phenotypes have been associated with therapy adaptation and resistance (H. Li et al., 2023). Yet, the evidence for the direct role of specific microbes or their metabolic products in mitochondrial reprogramming in TMZ-resistant GBM has not been provided yet. That is why it would be reasonable to consider the microbiome-mitochondria-TMZ resistance interaction as a biologically plausible but not fully verified mechanism (Ganesh et al., 2025; Koynova-Tenchov, 2026).

It is critical in regard to translational research. Instead of thinking that modifications in the microbiome and the metabolites produced by the microbiome would lead to improvement in the efficacy of the TMZ treatment, further research needs to focus on finding out whether there is an association between specific microbe types or their metabolites and a certain phenotype of mitochondria, responsiveness to the treatment, or drug resistance in GBM patients. Longitudinal studies incorporating information about the microbiome composition, the levels of microbial metabolites in the circulation, mitochondrial function, metabolic status of the tumor, immune phenotypes, and treatment responses would be essential in establishing whether these associations were causal or secondary effects of disease and treatment.

Collectively, this points to mitochondrial plasticity as a potential common mechanism underlying metabolic adaptation and GSC survival and therapy resistance and cancer recurrence as well as immune dysfunction in tumors, while the role of microbial cues in each of these is still being defined.

## 5 Immunometabolic reprogramming of the brain tumor microenvironment

Tumor metabolic reprogramming not only involves tumor cells but immune cells and stroma components in the tumor microenvironment (TME). For GBM, the metabolic state that includes hypoxia, nutritional depletion, changes in glucose and fatty acid metabolism, and inflammation can affect the efficacy of antitumor immunity and the adaptability and resistance of the tumor (Huang et al., 2025; Su et al., 2024). Metabolic mechanisms such as glycolysis, lactate, lipids metabolism, tryptophan, and glutamine are responsible for the metabolic status of tumor and immune cells (Vijayanathan and Ho, 2025). Hence, the TME is an ideal junction for tumor metabolism, immune cell metabolism, mitochondria, and microbes.

### 5.1 Innate immune regulation

Innate immune population is one of the main constituents of the GBM TME, and consists of TAMs, microglia, monocyte-derived macrophages, and MDSCs. The populations can be associated with the progression of tumor due to their participation in cytokine generation, metabolic programming, cytotoxic immunity inhibition, and immunosuppressive microenvironment creation (Nejadrostam et al., 2026). TAMs consist of different populations of microglia and monocyte-derived macrophages, which have particular metabolic and functional states that affect tumor behavior and patients’ outcomes (Solomou et al., 2024; Kloosterman et al., 2024).

Metabolism of myeloid cells is closely connected with cytokines generation, inflammation pathways, phagocytosis, and communication with tumor cells (Essakhi et al., 2024). MDSCs and other myeloid populations can additionally participate in immune suppression and decrease antitumor activity and efficacy of immunotherapeutic interventions (Li et al., 2025). In the context of the microbiome-mitochondria axis, these populations become a great opportunity for creating another interface, as microbial metabolites can modulate metabolism and mitochondrial activity of immune cells, and metabolism and mitochondrial state can affect immune response and inflammatory phenotype of TAMs and MDSCs. It should be determined whether gut microbial signature affects mitochondrial state in GBM-associated myeloid populations directly.

### 5.2 Adaptive immune responses

In GBM, adaptive immunity is marked by limited T-cell infiltration and significant dysfunction. There are only limited numbers of CD4+ and CD8+ T-cells compared to total infiltrating leukocytes, while Tregs might infiltrate into the TME and secrete immunosuppressive cytokines like IL-10 and TGF-β (Zhang et al., 2025; Xu et al., 2023). The immunosuppressive nature of TME is created by lack of neoantigens, activity of myeloid cells, and metabolic restriction due to arginase and IDO pathways (Woroniecka et al., 2018; Luning et al., 2025).

Limitation in nutrients, hypoxia, immunosuppressive metabolites, and signaling molecules from the tumor itself might cause dysfunction and exhaustion in T cells, which is seen as higher levels of CTLA-4, PD-1, and TIM-3 receptors (Akbari et al., 2026). Since activation and maintenance of T-cell function is contingent upon proper mitochondrial metabolism, microbiota-produced metabolites that change the supply of substrates to the body, redox state, or metabolic signaling have the potential to affect T-cell bioenergetics. Such an effect can be synergized by the nutrient deprivation, hypoxia, and immune suppressing metabolites that exist in the GBM tumor microenvironment and thus lead to T-cell dysfunction. Yet, there is not enough evidence for such a mechanism in humans.

### 5.3 Neuroinflammation and immune evasion

GBM-related neuroinflammation is characterized by orchestrated interplay between the cancer cells, microglia, macrophages, lymphocytes, endothelial cells, and inflammatory mediators. Cytokines like IL-6, IL-1β, and TNF-α are among factors that play a role in inflammatory signaling and TME remodeling (X. Li et al., 2024; Esparza-Salazar et al., 2025). It is worth noting that inflammation does not always lead to antitumor immunity; persistent inflammatory signaling and immunosuppression can coexist (Essakhi et al., 2024). Regulatory B cells and other immune-suppressive populations can suppress cytotoxic lymphocytes using IL-10, TGF-β, and other TME-derived mediators.

Immune checkpoints such as CTLA-4, PD-1, and PD-L1 belong to the most important regulatory pathways, but the low efficiency of checkpoint blockade therapy in GBM demonstrates the impact of the metabolic and immunologic milieu (X. Li et al., 2024). Exhausted immune cells can stay impaired in case of nutrient starvation, hypoxia, immunosuppressive metabolites, mitochondrial dysfunction, and suppressive myeloid cells. Metabolites produced by the microbiome can create this milieu by regulating inflammation and immune system on a systemic level. At the same time, tumor-related inflammation and the changes in host metabolism can affect the microbiome of the intestine. The interaction goes both ways, not only from the gut to the tumor; however, it is still necessary to identify the microbial signals controlling neuroinflammation and mitochondrial function in human GBM.

### 5.4 Metabolic communication among tumor, immune, and microbial components

The GBM TME can thus be conceptualized as a metabolically integrated network wherein there is competition between tumor and immune cells for metabolic substrates, and both respond to common metabolic cues. Increased consumption of glucose by tumor cells increases the generation of lactate, leading to acidification of the TME, suppression of effector T cells and NK cells, and induction of an immunosuppressive macrophage phenotype (Medikonda et al., 2025; J. Huang et al., 2026). Competitive utilization of glutamine, tryptophan, arginine, and other nutrients by immune cells is yet another mechanism of suppressing immune cells’ function. Metabolism of lipids, on the other hand, influences immune cells’ mobility and activity (Mao et al., 2026).

Microbial metabolism in the gut is the next aspect of this network of interconnected metabolic processes. Microbiome changes alter the production of SCFAs and other metabolites and microbiome-associated alterations in serine and glycine metabolism influence one-carbon metabolism and amino-acid availability (Green et al., 2024; Mangano et al., 2026). The impact of microbially derived metabolites on host metabolic and redox pathways implies effects of these metabolites on mitochondrial bioenergetics, oxidative stress, and metabolic adaptations (Koynova-Tenchov, 2026). These changes, in turn, may influence the metabolic status and functionality of both tumor and immune cells. However, the mechanisms through which these microbial metabolic signaling processes affect GBM-related cells have not been fully elucidated.

This interaction must be examined in the context of the full host-tumor system. Factors such as genetic predisposition, immunological condition, metabolism, medication intake, and other physiological conditions affect both tumor biology and microbiome, while the tumor itself impacts host physiology through inflammation and metabolism (Mangano et al., 2026). A possible point of integration may include mitochondria which react dynamically to nutrient availability, intermediate metabolites, oxidative status, and inflammation. Thus, the metabolites generated by the microbiota might impact the mitochondrial and metabolic status of GBM cells, tumor-associated macrophages, microglia, and lymphocytes with consequences for growth, stress resistance, functioning of immune cells, and immune tolerance. However, despite the emerging evidence of interactions between the metabolites produced by the microbiota and host metabolic and mitochondrial systems, there is not enough information to prove that changes in the gut microbiome induce the mitochondrial reprogramming in GBM cells and GBM-associated immune cells. Moreover, a direct causal relationship between microbiome, mitochondria, and GBM progression has not been proven in humans yet. Thus, this potential axis should be viewed as an emerging mechanism, which requires validation via longitudinal studies examining the changes in microbiome composition, metabolite profile, mitochondrial phenotype, immune cell states, and tumor biology (Ganesh et al., 2025; Green et al., 2024; Koynova-Tenchov, 2026) (**Figure 4**).

**Figure 4:**
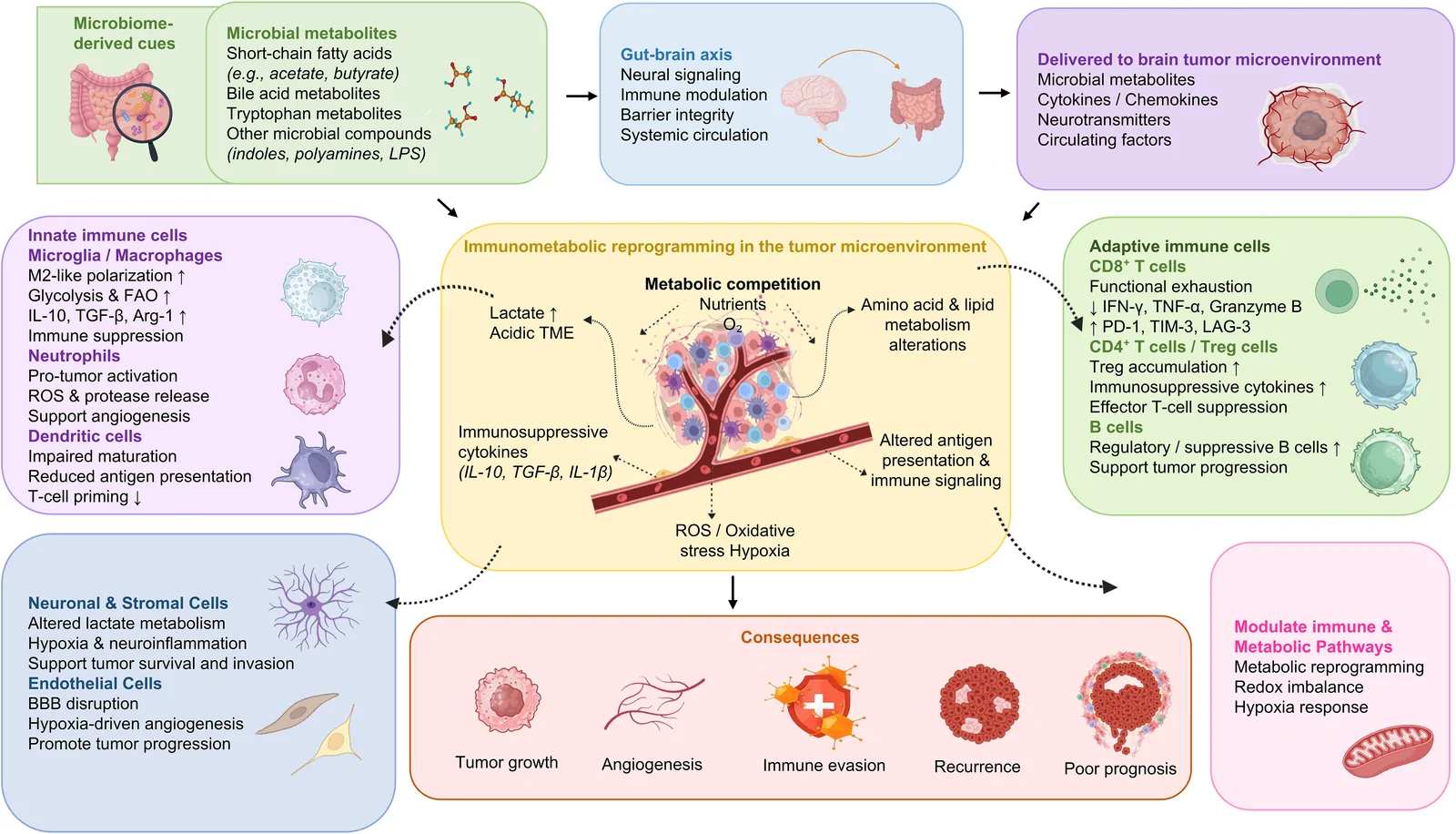
Microbiome-derived metabolites regulate the gut-brain axis and reshape the brain tumor microenvironment by altering cellular metabolism, redox balance, hypoxia, immune signaling, and metabolic competition. These changes influence tumor growth, angiogenesis, immune evasion, recurrence, and therapeutic response.

## 6 Clinical and translational implications

Even though more studies prove the relationship between the microbiome and the immune system, metabolism, and brain tumors, there is no clear understanding of the clinical importance of gut dysbiosis in glioblastoma (GBM). The current available evidence proves the effects of metabolites produced by microbes and related immune pathways in the tumor microenvironment. However, which microbiological factors are associated with the tumor growth, development, and response to treatment remains unknown. Therefore, although the microbiome-based markers become more widespread, they require further validation in well-characterized patients’ populations. The individual microbiomes’ uniqueness adds to the challenge of identifying the microbial signatures and emphasizes the need for personalized treatment approaches (Toumazi et al., 2025). On the other hand, one more research avenue related to the precision oncology is mitochondrial biology. Branched-chain amino-acid (BCAA) metabolism, oxidative phosphorylation (OXPHOS), reactive oxygen species (ROS) regulation, mitochondrial transfer, and mitophagy-driven cell death have become actionable cancer biology targets recently. While most of the mitochondria-targeting approaches have yet to pass from preclinical studies into the realm of investigation, continuous advancements in mitochondria vulnerabilities identification made them increasingly applicable for cancer therapy (Liang et al., 2025). Combining the above described dimensions may thus open additional possibilities for biomarker discovery and stratified cancer treatment (Liang et al., 2025; Ganesh et al., 2025).

### 6.1 Microbiome-derived biomarkers

The gut microbiome represents a promising source of biomarker discovery since it is possible to evaluate not only the microbial community but also its metabolites in feces, blood, and other biofluids. It should be noted that the microbiota constitutes an actively metabolizing ecosystem that produces or transforms various bioactive molecules, such as SCFAs, secondary bile acids, and other metabolites associated with metabolic and inflammatory status of the host (Mangano et al., 2026). The evaluation of microbial and metabolic signatures can provide a reflection of systemic biological states that could complement data obtained from tumors. Current clinical research revealed distinctive gut microbial signatures in GBM patients in comparison with healthy individuals, providing the possibility of developing non-invasive biomarkers based on microbial signatures. It has been found that a set of fecal microbes represented by Fusobacterium, Escherichia/Shigella, Ruminococcus gnavus group, Lachnospira, Akkermansia, and Parasutterella was able to discriminate patients with GBM from healthy controls at the level of approximately 0.80 of area under the curve (Jiang et al., 2024).

It is also possible to point out the contribution of genetic epidemiology to the discovery of the specific microbiota that might be associated with the risk of GBM. Mendelian randomization analysis has revealed some taxa, including Anaerostipes, Faecalibacterium, Prevotella 7, and Ruminococcaceae, that can be potential protective factors for GBM, but these results should be validated for being used as clinical biomarkers. It has been also suggested that Bifidobacterium infantis and Coprobacillus cateniformis are associated with the increased and decreased risk of GBM, correspondingly (Mohamed et al., 2025; Gao et al., 2026). The protective effect of Anaerostipes can be explained by its immunomodulatory activity in the context of colorectal cancer, which indicates that some microbiota members can affect tumor development through immune system (Mohamed et al., 2025).

Microbial metabolites can also represent a complementing source of biomarkers. Metabolic profiling of GBM patients and controls revealed several metabolites derived from both the microbiome and the host organism. The concentrations of tryptophan, serotonin, indole-3-propionic acid, indole-acetic acid, and phenylalanine and tyrosine-derived compounds such as 4-ethylphenylsulfate and phenol sulfate were decreased in GBM, while the concentrations of metabolites involved in detoxification, carnitine catabolism, and serotonin degradation, including hippuric acid, indoxyl-glucuronide, carnitine, TMAO, and 5-hydroxyindoleacetic acid, were increased (Khalife et al., 2025). Thus, circulating metabolites may be used to measure the alterations in the host-microbe interaction and serve as complementary metabolic biomarkers.

Recently, additional metabolites associated with the microbiome were identified. A causal mediation analysis combining the data on gut microbiome, cerebrospinal-fluid metabolites, and GBM risk revealed positive associations between Coprobacillus cateniformis and GBM and negative associations between GBM and S-1-pyrroline-5-carboxylate with potential mediating effect on the association between C. cateniformis and GBM (Gao et al., 2026). Thus, the presented results represent an example of a microbiome-metabolite signature potentially valuable for diagnostics and understanding the disease mechanisms, but requiring further clinical validation.

Biomarkers derived from the microbiome may also have potential application for the prognosis of GBM and response to treatment. In a prospective phase I/II study of GBM patients treated with the anti-PD-L1 regimen, enrichment in the bacteria predominantly belonging to the Bacillota (Firmicutes) phylum was noted in the group of patients with longer overall survival, while Bacteroides thetaiotaomicron was enriched in tumors characterized by a low immune environment score (Weathers et al., 2025). Therefore, the gut microbial composition may serve as a prognostic marker if used in combination with immune properties of GBM.

In turn, experimental investigations showed that the microorganisms of the gut and their metabolites are different in the case of temozolomide-sensitive and non-sensitive glioma models. Microbiome and metabolomics analysis revealed that the particular bacteria and metabolites were linked to the pharmacodynamic reaction on temozolomide; furthermore, the gut microbiome depletion decreased the efficiency of temozolomide and changed the macrophages and CD8α^+^ T-cell infiltration (Hou et al., 2023). Moreover, it was recently revealed that some plasma metabolites associated with the gut microbiome were correlated with the GBM state and chemoradiation treatment (Khalife et al., 2025). However, the former is experimental evidence, while the latter one is early clinical data.

However, it is important to underline that measuring the microbiome is an independent parameter and it should be done in combination with other factors. Nowadays, GBM metabolomics is carried out in relation to the tumor tissue, biofluids, and animals, including the metabolic signatures of stemness, IDH mutation, and MGMT promoter methylation (Costantini et al., 2025). Thus, combining the microbial composition with the circulating metabolites and molecular signatures of tumors could bring more information than separate taxonomic analysis. Therefore, it can be stated that microbiome-metabolite-tumor signatures may be more valuable for diagnosis, prognosis, and treatment-response prediction than individual microbial taxa or metabolites.

There are several limitations of the approach that need to be addressed before it can be put into practice. Firstly, microbiome composition is associated with diet, medication, antibiotics intake, geographical location, age, lifestyle, co-morbidities, and other factors related to patients’ health status. Secondly, despite the fact that a connection between some particular parameter and cancer exists, it is unknown whether this parameter plays a causal role in the development of cancer or is just a marker of this disease or therapeutic treatment. Therefore, microbiome-derived taxa and metabolites may be considered as candidate biomarkers but not yet clinical biomarkers of cancer.

### 6.2 Mitochondrial biomarkers

Secondly, mitochondrial phenotypes represent a group of biomarkers of potentially useful information since they reflect the metabolic and stress-adaptive status of the tumor cell. Several parameters concerning the oxidative phosphorylation system (OXPHOS), ROS production, mitochondrial transport, mtDNA and mitophagy could provide insight into tumor biology and therapy sensitivity (Liang et al., 2025). The changes in the copy number (CN) of mtDNA have been studied extensively in GBM patients. Changes in mtDNA CN could affect the function of the mitochondria, oxidative metabolism and redox state. However, either increased or decreased mtDNA CN can influence OXPHOS and ROS production in different cell types. These changes could interact with the genetic and environmental characteristics of TME and affect tumor progression and therapy (Yusoff et al., 2025). The above data suggest that there is potential for further study of the mtDNA-based parameters as candidates for biomarkers of GBM, although their value needs to be proved prospectively.

Thirdly, mitochondrial respiratory activity presents a possible candidate for the role of biomarker and therapy target. Abnormalities in the activity of the mitochondrial electron transport chain (ETC) can influence ROS production and cell metabolism. Dependence of tumor cells on OXPHOS creates metabolic vulnerabilities which could be potentially used for tumor therapy (Baharuddin and Yusoff, 2025). Instead of viewing mitochondrial dysfunction as one dichotomy, further biomarker research will benefit from the assessment of several aspects at once, including respiratory activity, mitochondrial mass, potential of the membrane, ROS production, mtDNA and mitochondrial quality control.

Fourthly, mitochondrial transfer represents an extra dimension of tumor metabolic plasticity. Horizontal transfer of mitochondria has been shown in various types of cancers and allows acquisition of functional mitochondria by the tumor cells which results in changing of their metabolic ability. The above data contributes to the new interest in mitochondrial metabolism, in spite of the classical role of aerobic glycolysis in cancer metabolism (Brisudova et al., 2025a). The above fact suggests that the strategies directed against OXPHOS and ETC could be applicable to specific tumor subsets, although the criteria for selecting patients are not clear yet.

Accordingly, the approach to mitochondrial biomarker development should go beyond the simple measurements and should address the question whether mitochondrial signatures can distinguish relevant tumor states. Integration of the mitochondrial measurements with the tumor genotype, metabolomics and treatment history will provide a more informative way for patient stratification.

### 6.3 Integrated microbiome-mitochondria signatures for precision neuro-oncology

Integration of information on microbiome and mitochondria allows for the development of more powerful biomarkers. While profiling of the microbiome gives the picture of its systemic composition and metabolic profile, measurements of mitochondrial functions can show the functional metabolic state of the host cells. Thus, the integration of the two types of information could allow to connect upstream systemic signals with the downstream cellular phenotypes. At present, efforts in the field of GBM treatment are focused on the exploration of the role of the microbiome in GBM and development of targeted therapeutic approaches. Potential methods include manipulation of the microbiota via probiotics, manipulation of the microbial population as a whole, FMT, and modulation of the interactions between microbial signals and antitumor immunity. Moreover, other possible methods for overcoming treatment barriers, including utilization of bacterial toxins that promote the penetration of the BBB were considered (Toumazi et al., 2025). However, all these approaches are still experimental and need further validation for their effectiveness and safety before implementation in clinical practice (Toumazi et al., 2025).

The multi-omics approach could be the methodological basis for the development of integrated signatures. It includes genomics, transcriptomics, proteomics, metabolomics, and microbiome profiling. Thus, it allows for characterizing the relationship between the tumor genotype, host metabolism, immunity, microbial population, and therapy resistance. Despite the rapid progress of the technology, there are no approaches aimed at the systematic integration of these data in the case of neuro-oncology specifically (Toumazi et al., 2025; Jiang et al., 2025). Moreover, community structure of microbes might give some additional prognostic information. For example, there was established an association between the relative abundance of Bacteroidaceae and Lachnospiraceae and GBM outcomes because of the difference in these microbial populations related to DNA-repair activity, resistance, and immunosuppression. These data might indicate that microbial population structure might be informative, however, more investigations are needed. Besides, there are several mitochondria-related processes including BCAA metabolism, OXPHOS, ROS regulation, mitochondrial transfer, and mitophagy that could serve as complementary information about the metabolic state of the tumor (Liang et al., 2025). At the same time, other therapeutic platforms such as exosomes and oncolytic viruses are also being considered for GBM.

## 7 Therapeutic opportunities targeting the microbiome-mitochondria axis

Insights into the interactions between microbial metabolism, mitochondria, tumor metabolism, and the immune microenvironment have yielded several promising therapeutic approaches to GBM. These comprise microbiome, mitochondrial, metabolic, and immunometabolic approaches, as well as rational combination treatments. However, the literature regarding these treatments is diverse. Some of the information is obtained from GBM-specific experimental models or patient populations, while other mechanisms are known in relation to non-GBM tumors, neurological disorders, and experiments that have not been done within the field of neuro-oncology. Therefore, microbiome-, mitochondrial-, and metabolism-based treatments for GBM at this time should be regarded as investigational only, with their rationale based on preclinical research and novel patient-level data (Medikonda et al., 2025; Toumazi et al., 2025; Liang et al., 2025).

### 7.1 Microbiome modulation strategies

Microbes and their metabolites could modulate host metabolism, host immune system, and transcriptional and epigenetic programs, thus opening opportunities for targeted microbiome manipulation with the help of dietary manipulation, probiotics, prebiotics, and FMT as an auxiliary tool to cancer treatment (Hajjar et al., 2026). In GBM, the above-mentioned therapies might affect systemic immune tone and metabolism of the metabolites influencing the tumor microenvironment. One of the potential interfaces of therapy can be the SCFAs (acetate, propionate, and butyrate). All three SCFAs could influence immune cells’ function and epigenetics (through histone deacetylase inhibition), as well as maintain BBB integrity and modulate CNS immune response (Dey et al., 2026). Still, the effects of individual SCFA might be context dependent, and increasing or decreasing concentration of certain microbial metabolite without knowledge of its target cell is unlikely to result in the beneficial effect. Thus, manipulating of microbiome must be aimed at restoring of favorable metabolic environment rather than increasing of microbial diversity.

Dietary manipulations present one of the easiest ways of microbiome modification. Higher dietary fiber consumption is linked to the positive effects in immune checkpoint inhibition in observational studies, while experimental evidence shows that diet is capable of modulating the numbers and functional state of antitumor CD8+ T cells after immunotherapy (Hajjar et al., 2026). As prebiotics also affect microbial composition and production of metabolites, they are also studied regarding their potential in improving immune function and influence on cancer-associated processes (Green et al., 2024). Still, the optimal composition of diet, timing, and population of patients, who are suitable for such interventions, are unknown, so dietary interventions cannot yet be considered established GBM therapies.

Another potential way of manipulating of microbiome is the use of probiotics. The mechanisms of action of probiotics include modulation of host immune system, production of neuroactive compounds, inhibition of pathogens, and improvement of intestine barrier function (Sánchez et al., 2016; Chandrasekaran et al., 2024). Still, traditional probiotic interventions suffer from one major disadvantage - the introduction of small number of certain bacterial strains to the organism is unlikely to produce predictable and stable changes in resident microbiome. They depend heavily on the microbial composition of recipient, diet, treatments, and host physiology.

FMT represents another way of modifying the microbial composition. It is suggested to act as an adjuvant to immune checkpoint inhibition through the modification of microbial composition, metabolites’ profile, and antitumor immune response (Yang et al., 2024). Still, successful outcomes of such intervention depend on the compatibility between donor and recipient, as well as the extent and sustainability of microbial engraftment. Most importantly, the successful engraftment doesn’t always correlate with therapeutic efficiency. Regarding the microbiome-mitochondria axis in particular, one of the priorities in future will be the identification of the target of the manipulation instead of mere manipulation of microbial composition.

The available scientific evidence supporting microbiome modulation therapy for GBM is still in its infancy and has to be distinguished from scientific evidence gained through research in other cancers. Diet and prebiotics have been shown to change the microbiome composition and metabolism. However, there is no proof that they affect the GBM outcomes. Probiotic effects depend on many different factors: the initial state of the microbiome, diet, simultaneous treatment, and patient physiology, thus making it impossible to predict therapeutic outcomes. In turn, fecal microbiota transplant (FMT) has been shown to change the microbiome and immune response in other cancers. But the scientific evidence of FMT’s positive effect on the GBM is still weak (Yang et al., 2024; Liu Y et al., 2025).

### 7.2 Mitochondria-targeted therapeutics

The therapeutic benefits that mitochondrial plasticity can have also exist. There are several ways for mitochondria to move from one cell to another, which include mitochondrial transport through extracellular vesicles, excretion/uptake of free mitochondria, and intercellular bridges. These processes can influence cellular bioenergetics and stress responses, and thus there has been an increasing interest in cancer biology regarding these phenomena (Liang et al., 2025; Chung et al., 2025). Mitochondria can serve as a therapeutic target in GBM through exploiting dependence of tumor cells on OXPHOS, substrate utilization, mitochondrial redox state, or mitophagy. Being able to switch metabolism between glycolytic and oxidative modes, tumor cells can exhibit compensatory metabolic reprogramming upon targeting a certain metabolic pathway. Such adaptability highlights the importance of identifying metabolically sensitive tumor states.

Mitophagy can be viewed as a possible therapeutic target in this case. Elevated levels of mitochondrial turnover can enable tumor cells to sustain functional mitochondria under metabolic and therapeutic stresses, making dysfunction of mitophagy mechanisms a way to impair tumor-cell viability (Brisudova et al., 2025b). Yet, since normal neurons and other brain cells heavily rely on mitochondrial functioning, targeting of mitochondrial metabolism involves the risk of neurological side effects. The metabolic plasticity of GSCs is illustrative of this problem. Metabolism-type GSCs are capable of sustaining high levels of ATP synthesis and oxygen consumption, but can switch to glycolytic pathway with enhanced lactate production in response to hypoxia or inhibition of OXPHOS. Such reversibility shows that GBM cells can flexibly shift the distribution of their bioenergetic dependence depending on external stresses (Storevik et al., 2026). On the other hand, there are reports showing that energy generation of GSCs remains almost constant following inhibition of glycolysis or OXPHOS, with elevated mitochondrial reserve being associated with radioresistance (Vlashi et al., 2011). Altogether, these results show that mitochondrial targeting should consider tumor metabolic heterogeneity instead of the assumption of uniform dependence on OXPHOS in GBM.

While these results suggest mitochondria-dependent pathways as promising targets for GBM treatment, the existing data are primarily preclinical. However, the metabolic flexibility of GBM and the reliance of normal neurons on mitochondria limit the therapeutic window. Thus, targeting OXPHOS, mitophagy, and other mitochondrial mechanisms might result in compensation of metabolism in cancer cells, along with damaging effects on normal brain tissue. Hence, future therapies targeting mitochondria should aim at selectively targeting vulnerabilities in tumor cells and use pharmacodynamic markers of mitochondrial dependence, including OXPHOS activity, mitochondrial reserve capacity, ROS levels, and mitophagic phenotypes.

Finally, mitochondrial transfer complicates matters. While mitochondrial exchange has been shown in a number of experimental cancer models, most of this evidence comes from *in vitro* experiments or tumor models that have been experimentally deprived of their mitochondria. Hence, the role of endogenous transfer of mitochondria from the TME to tumor cells has not been fully elucidated (Watson et al., 2023). This aspect is especially relevant when speaking about targeting of mitochondrial transfer. Metabolic adaptation contributes to chemoresistance of tumor cells. Analysis of metabolome in TMZ-resistant GBM cells has revealed metabolic alterations in glycolysis, TCA cycle, fatty acid metabolism, and amino acid metabolism, confirming metabolic rewiring occurring in the process of resistance acquisition (Tomar et al., 2025). In this manner, mitochondrial targeting can serve as a biological rationale for therapy of metabolically reprogrammed GBM, or treatment-resistant GBM, but its clinical relevance needs to be proven. Given that mitochondrial functions are essential for proper functioning of healthy neurons, future studies should focus on selective targeting of tumor-dependent mitochondrial functions.

### 7.3 Immunometabolic interventions

Given the intimate interaction between metabolism of tumors and immune cells, approaches to alter immunometabolism may be useful to add to existing microbiome and mitochondrial therapies. GBM cells are in competition with immune cell infiltrates for glucose, amino acids, lipids, and other metabolic nutrients, and tumor-derived metabolites, like lactate and kynurenine, may suppress immune cell functionality (Vijayanathan and Ho, 2025; L. Yang et al., 2025). Inhibition of the metabolism mentioned above, namely, glycolysis, lactate production/signalization, glutaminolysis, and amino-acid metabolism, would not only inhibit tumor metabolic fitness but also alleviate the metabolic limitations for antitumor immune cells (L. Yang et al., 2025). The problem with that is that the processes above are essential for functioning of activated immune cells, making selectivity of therapeutics crucial.

The metabolic remodeling that goes together with GAM polarization may provide another interesting target for therapy. Interference with the described processes might enable the transformation of macrophages and microglia into more antitumor phenotypes and reduce their contribution to tumor progression (Gao et al., 2025). Conceptually, the approach above is quite interesting because of its focus on functional aspects of TME instead of tumor cells exclusively. Metabolic activity of mitochondria also represents a possible intervention point for the described immunometabolic system. Both GBM cells and immune populations are different from each other in terms of glucose, fatty acid, amino acid, and mitochondrial metabolic activities. The inhibitory action of etomoxir against carnitine palmitoyltransferase 1 used in conjunction with TMZ has led to larger ATP production reduction in comparison with single treatments in experimental settings (Essakhi et al., 2024). It should be said that the approach represents the broader concept of combining conventional therapy with metabolic interventions despite the need to test individual metabolic inhibitors’ specificity and translational safety. Thus, the key point is not only in suppression of tumor metabolism but in alteration of the competitive metabolic activity in favor of antitumor immune response. This issue becomes especially relevant given the commonality of metabolic pathways in GBM.

The results showing synergy between etomoxir and TMZ constitute a proof-of-concept example of metabolic co-targeting for GBM; however, such an approach is not necessarily indicative of clinical benefit. In general, approaches that target glycolysis, oxidative fatty-acid metabolism, glutamine utilization, lactate signaling, or amino acid metabolism have the potential to impact both tumor and immune cells due to their presence in multiple cell types within the TME. Consequently, any success of such an approach would require high selectivity for the metabolic dependency of tumor cells without impacting the metabolic needs of antitumor immune cells and normal brain tissue.

### 7.4 Combination therapies

The interconnections between the microbiome, mitochondria, tumor metabolism, and the immune system make an argument for studying combination therapies for GBM. Interventions targeting the microbiome might influence the production of microbial metabolites or the processes of metabolism and immunological signaling of the host, while the inhibitors of mitochondrial or metabolic pathways can impair the ability of tumor cells to compensate for their metabolic challenges. These therapies are conceptually applicable to GBM since metabolic plasticity can allow tumor cells to escape from the inhibition of individual bioenergetic pathways.

In preclinical experiments, OXPHOS, mitochondrial ROS, mitochondrial transport, and mitophagy were found to be potential targets of therapy that might be used in combination with standard therapy or with metabolic interventions (Liang et al., 2025). Other techniques, such as the inhibition of VDAC1 and approaches targeting tumor hypoxia, were also explored in preclinical experiments (Chung et al., 2025). Nonetheless, this research suggests a therapeutic approach but does not prove its effectiveness since the optimal combination and duration of therapy are unknown and metabolism might compensate for a targeted mitochondrial pathway.

Studies in human GBMs further reinforce the rationale for biomarker-driven combination approaches, though the therapeutic value of microbiome modulation has yet to be demonstrated. In a clinical study combining metagenomics in feces with tumor transcriptomics, genomics, and microenvironmental characterization, gastrointestinal bacteria features were found to be associated with tumor immune infiltration, features, and clinical outcome (Weathers et al., 2025). It shows that microbiome and tumor features can be complementary in terms of providing biomarkers for treatment selection; however, it is not an indication of the positive effect of microbiome modulation on the treatment outcome or immunotherapy response.

Future combination therapies have to rely on specific biological weaknesses rather than empiric combinations. For instance, patients with a reproducible microbial-metabolic profile can be evaluated for microbiome or metabolite-driven interventions, while tumors with a specific mitochondrial dependence can be assessed for mitochondrial-targeted treatments. Combining microbiome composition, circulating microbial metabolites, mitochondrial phenotypes, tumor metabolism, immune cell profile, molecular subtype, and treatment history can lead to identification of biologically plausible combinations. It will require validation of the signatures to determine whether they predict response, contribute to treatment resistance, or can be modified through therapy (**Figure 5**).

**Figure 5:**
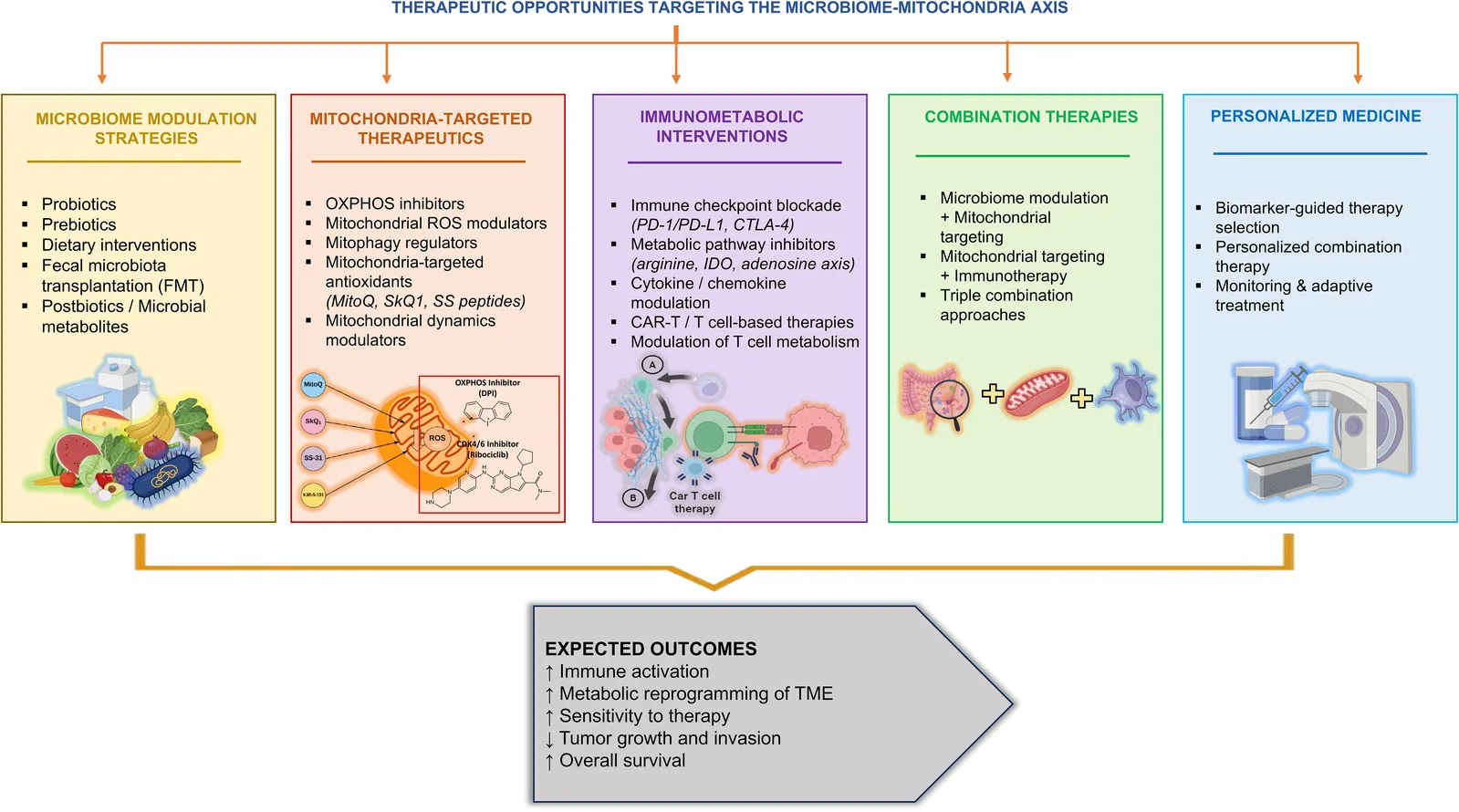
Therapeutic targeting of the microbiome-mitochondria axis includes microbiome modulation, mitochondrial therapeutics, immunometabolic inhibition, combination strategies, and personalized medicine. These approaches may enhance immune activation and treatment sensitivity while reducing tumor growth, invasion, and therapy resistance.

### 7.5 Personalized and precision medicine

The considerable variability both within the GBM metabolic signature and gut microbiota indicates that the universal approach based on the microbiome-mitochondria interaction might be inefficient. However, future individualized treatment methods may include personalized identification of microbiome profile, circulating metabolites, tumor metabolic profile, immune cell phenotype, molecular GBM subtypes, and history of therapy (Elkrief et al., 2025; Chung et al., 2025; Liang et al., 2025). These strategies are especially applicable to immunotherapy. While tumor immune infiltration can determine response to PD-L1 blockade, the number of infiltrating cells may not be sufficient to predict increased survival in some patients. Thus, the mesenchymal GBM subtype, with certain immune characteristics, may be a useful parameter for identifying patients who benefit more from PD-L1 therapy in the clinical setting outlined by Weathers et al. (2025). Thus, the treatment response is determined not by the presence of a particular biomarker, but by the interaction between the tumor genetic profile, immune cell phenotype, metabolism, and other factors.

Therefore, future treatment algorithms could be developed using information about the patient’s genome, metabolomics, microbiome, immunology, and geographical peculiarities to formulate an individual treatment regimen. Longitudinal sampling becomes especially important because both tumor metabolic activity and microbiome composition change over time under the effects of therapy. Therefore, the initial profile of the patient’s microbiome may become less useful than the dynamic interactions among microbial profile, metabolites, mitochondrial status, and immune characteristics. Various factors are necessary before the implementation of microbiome-mitochondria precision therapy in neuro-oncology practice, including standardization of biomarkers, a reproducible method of analysis, longitudinal samples, appropriate tumor and host phenotyping, clinical testing, and proving that the use of biomarker-driven intervention improves treatment results. It should be noted that currently the microbiome-mitochondria precision therapy paradigm is still in development (**Table 2**).

**Table 2:** Therapeutic targeting of the microbiome-mitochondria axis in neuro-oncology.

Therapeutic strategy	Specific intervention/example	Microbiome-mitochondria connection	Potential therapeutic outcome	Evidence base/translational status	Key references
Microbiome modulation • Microbial metabolite targeting	Dietary interventions, prebiotics, probiotics, synbiotics, FMT SCFAs, particularly butyrate; tryptophan-derived metabolites	Modifies microbial composition and metabolite production, potentially influencing host metabolism and mitochondrial signaling Microbial metabolites regulate mitochondrial metabolism, redox signaling and immune-cell function	Immune and metabolic reprogramming; improved treatment responsiveness Modulation of tumor growth, inflammation and immune surveillance	Preclinical; emerging clinical evidence Mainly preclinical	(Chen et al., 2025; Green et al., 2024)
Mitochondrial targeting • Mitochondrial quality control	OXPHOS/ETC inhibition, mitochondrial ROS and metabolic targeting Mitophagy and mitochondrial dynamics/transfer	Intersects with microbial metabolite and nutrient availability Mitochondrial adaptation may be influenced by cellular and metabolic signals	Impaired tumor bioenergetics and increased metabolic stress Impaired tumor bioenergetics and increased metabolic stress	Preclinical/early translational Preclinical	(Liang et al., 2025; Chung et al., 2025; Liang et al., 2025; Storevik et al., 2026)
Immunometabolic targeting	Glycolysis, glutaminolysis, lactate and myeloid-cell metabolism	Microbial metabolites can influence tumor-immune metabolic interactions	Reduced immunosuppression and enhanced antitumor immunity	Preclinical/early translational	(Gao et al., 2025; Yang et al., 2025)
Combination therapy	Microbiome modulation + immune checkpoint blockade/metabolic therapy	Simultaneous manipulation of microbial, immune and metabolic pathways	Potential to overcome treatment resistance	Emerging clinical/preclinical	(Weathers et al., 2025; Liang et al., 2025; Chung et al., 2025)
Personalized approaches	Microbiome + metabolomic + tumor/immune profiling	Identifies patient-specific microbial and mitochondrial/metabolic phenotypes	Treatment stratification and individualized therapy	Early translational/future	(Chen et al., 2025; Weathers et al., 2025; Green et al., 2024)

## 8 Emerging technologies and future directions

The ever-growing complexity of the microbiome-mitochondria-immune-tumor network necessitates the use of novel technologies that would allow one to analyze biological processes occurring on several molecular and spatial levels. Technologies such as high-throughput sequencing, metabolomics, single-cell and spatial analysis, and artificial intelligence (AI) have recently emerged as complementary ways to study this complexity. Their combination could be used to differentiate changes intrinsic to tumor cells from those related to the microenvironment and systemically occurring, to elucidate metabolic interactions between the microbiome and host, and to determine molecular hallmarks of tumor growth and treatment resistance.

### 8.1 Multi-omics and systems biology

Despite the progress in surgical interventions, radiotherapy and TMZ-based chemotherapy, GBM continues to exhibit suboptimal clinical results, with the median survival usually staying around 12–15 months (Marenco-Hillembrand et al., 2020). Conventional genomic and molecular profiling allows to discover important clinically relevant factors, including IDH mutations, MGMT promoter methylation, and EGFR alterations, but any single molecular layer reflects merely a small part of the underlying biological diversity that determines progression and responsiveness to therapy. The integrated multi-omics methods which include genomics, transcriptomics, epigenomics, proteomics, metabolomics, and non-coding RNAs would enable researchers to create a more complete picture of tumor biology. Genomic profiling would enable us to identify recurring mutations and molecular aberrations, whereas transcriptomic profiling would help determine the transcriptional status, splicing and fusion events (Kakde et al., 2026). Metabolomics is especially valuable for the microbiome-mitochondria hypothesis since metabolites are the end points of functionality that link microbial activities with cellular physiology of the host organism. Metabolite profiling of tissue and biofluid samples would allow to determine carbohydrate, amino acid, lipid, nucleotide, and other metabolite alterations associated with tumor progression and resistance to the treatment (Costantini et al., 2025). In contrast to genomic alterations, metabolomic profile can also reflect dynamic effects associated with treatment, nutrition, and disease progression.

Thus, one of the promising ways forward is to combine microbial metabolites with host and mitochondrial metabolites. This combination would help researchers to separate the metabolites produced and/or modified by the microbiota from metabolic alterations observed within tumors and immune system cells. Integration with mitochondrial measurements, including OXPHOS activity, ROS signature, mtDNA status, and mitochondrial quality control pathways, would help to reveal links between systemic microbial signaling and cellular mitochondrial phenotype. Longitudinal systems biology approach would allow to add temporal dimension, collecting molecular features before the treatment, during therapy, at the point of response or progression, and at the point of recurrence. Thus, integration of the microbiome, metabolomics, mitochondrial measurements, and clinical data would allow to identify molecular signatures associated with different disease states and treatment responses (Dono et al., 2025). However, such approaches need to focus on causality and reproducibility, not just on generation of growing list of molecular features associated with each other.

### 8.2 Single-cell and spatial omics

Bulk molecular profiling can mask the cellular heterogeneity that exists in GBM. Single-cell RNA sequencing (scRNA-seq) is therefore an increasingly useful technique for uncovering transcriptional states at single-cell resolution and identifying distinct malignant and non-malignant populations in the tumor microenvironment (Suvà and Tirosh, 2019). In gliomas, single-cell analysis has been used to identify proliferative malignant populations, stem-like states, mesenchymal-like populations, neuron-like tumor-cell states, and a variety of immune populations with different molecular and functional properties (Fu et al., 2025). In the case of the microbiome-mitochondria hypothesis, the significance of single-cell techniques is to establish which cell populations are sensitive to microbial or metabolite signaling. A metabolite from microbes found in the circulation might affect tumor cells, microglia, macrophages, endothelial cells, astrocytes, and infiltrating lymphocytes in different ways. It will be difficult to establish this using bulk analysis; however, single-cell profiling can establish which cells show cell-specific transcriptional and metabolic responses.

Spatial transcriptomics is another way to obtain more information by maintaining the anatomical localization of these cellular states. GBM harbors different niches, such as hypoxic, perivascular, invasive, and immune-enriched niches, which may show different metabolic states and communication networks (Lemoine et al., 2025). By performing spatial profiling, it becomes possible to establish whether certain mitochondrial or immune phenotypes exist predominantly within certain tumor niches. Such anatomical specificity is important due to the fact that the microbiome signal is not likely to affect all parts of the tumor equally. The presence of oxygen, the concentration of nutrients, access to blood vessels, immune cell density, and the type of cells may impact the response to a metabolite from circulation. Spatial omics might therefore indicate whether microbiome-related signatures cluster in certain niches of the tumor.

In general, spatial profiling will allow the understanding of the interaction between tumor cells and microenvironment and the role of these interactions in survival, invasion, migration, and resistance to treatment (Whittle et al., 2025). Coupling of spatial transcriptomics with metabolomics, mitochondrial phenotyping, and imaging can create a map of the microbiome-metabolite-mitochondria-immune system in individual tumors.

### 8.3 Artificial intelligence and computational modeling

The integration of data from the microbiome, multi-omics, imaging, pathology, and clinical fields results in very complex high-dimensional and heterogeneous datasets that are hard to analyze using traditional univariate or single-modality strategies. Artificial Intelligence (AI) in the form of machine learning (ML) or deep learning (DL) offers a computational tool for capturing non-linear associations between microbial abundance, circulating metabolites, mitochondrial performance, tumor molecular signature, immune cells, imaging features, and treatment outcomes. Recent reviews have noted the increasing use of AI for multi-omics, radiomics, and clinical data analysis in the case of glioblastoma (GBM), especially for molecular classification, prognostics, biomarkers identification, and treatment stratification (Morello et al., 2025; Abuhassan et al., 2026).

Predictive modeling can be considered the most immediate application of AI in this field. One can develop supervised learning methods capable of predicting clinically relevant endpoints, including overall survival, risks of progression, relapse, treatment response, and therapy resistance based on microbial, metabolic, mitochondrial, molecular, radiological, and clinical parameters. Time-to-event models can include information on survival endpoints, while classification or regression models can estimate the likelihood of treatment response or progression. On the other hand, unsupervised and representation learning methods can help identify previously unknown patient subgroups that display different microbiome-metabolite-mitochondrial phenotypes. In the case of GBM, a recent multimodal framework involving MRI, histopathological images, and transcriptomics has shown the potential of AI-based models in improving risk stratification compared to single-modality models, highlighting the role of combined biological and imaging data in predictive models (Zhao et al., 2025). However, such models must be viewed only as research tools until they can be proven reproducible and clinically useful in independent patient cohorts.

Another key area where the microbiome-mitochondria axis shows promise is multimodal data integration. Instead of treating the abundance of microbes, metabolite levels, mitochondrial traits, tumor genotyping, immune cell types, and imaging features as separate variables, AI models might incorporate all these information layers into one patient representation. For instance, a future AI model could integrate taxa and microbial function in the gut with microbial metabolites in circulation, mitochondrial DNA, oxidative phosphorylation, reactive oxygen species, mitophagy, tumor molecular subtype, immune cell types, MRI-derived features, and medical history. Various ways of multimodal integration, like feature-level, representation-level, and decision-level fusion can be applied depending on the data structure and availability. This approach may aid the discovery of composite signatures that cannot be found using traditional univariate analysis and could offer a more biologically relevant way of patient stratification and treatment response prediction (Morello et al., 2025; Abuhassan et al., 2026).

The problem of explainability is especially important when AI models are going to serve as biological interpreters or make any kind of recommendations for clinical practice. Even a high-performing DL model can make correct predictions while being unaware of which biological and/or clinical features were important for these predictions. In the scope of microbiome-mitochondria research, the problem of explainability is relevant due to the fact that an important predictor of an outcome in statistical sense does not necessarily mean that it is a mechanistic determinant of this outcome. The following techniques of making AI models more understandable include feature-attribution methods, perturbation-based approaches, gradient-based methods, model-specific explanation methods such as SHAP, etc. Increasing numbers of studies on glioma imaging have shown growing tendency of using XAI techniques as a *post hoc* analysis after deep learning models’ training for grading tumors, predicting biomarkers, localizing tumor foci, assessing intratumoral heterogeneity, and analyzing prognosis (Ayaz et al., 2025). However, caution is needed when interpreting *post hoc* explanations since the importance of features identified by a model does not mean their causal relation. Ideally, AI-derived associations should lead to a generation of mechanistic hypothesis that could then be tested experimentally.

Such progress also opens up possibilities for AI-assisted clinical decision-support systems in neuro-oncology. Rather than substituting clinical decision-making, AI tools would possibly be used to estimate individual probability of disease progression, disease recurrence, therapy response or resistance based on longitudinal information about a patient’s condition. Using microbiome-mitochondria framework, a decision-support system would be able to incorporate a patient’s microbiome profile, microbial metabolites circulating in his/her blood, mitochondrial biomarkers, tumor characteristics on molecular level, radiological data, immunological information, treatment history, and many more clinical parameters in order to develop a patient-specific risk profile. In future, such information could be utilized by clinicians for choosing patients requiring close observation, participants of clinical trials, and rational combination of microbiome-oriented, metabolic, mitochondrial and immunological therapies. At present, all these applications are under research, and the use of AI outcomes in clinical decision making needs to be prospectively tested in order to establish if model-driven decisions could be associated with better clinical outcomes.

Real-world implementation and validation is still one of the biggest problems. AI algorithms designed using retrospective datasets would perform quite well in their training and test populations but poorly in geographically, clinically or technically different population. Sources of variability would include differences in sequencing technology, image acquisition parameters, sample preparation protocols, microbiome profiling methods, dietary intake, medication usage, antibiotics usage, tumor biopsy procedures, medical care and EHR structure. Moreover, additional sources of variability would be class imbalance, missing data, batch effects, overfitting and information leakage from training data to validation dataset. Concerning microbiome-related models, high dimensionality and compositional nature of microbial data make feature selection and validation even more crucial. Therefore, a robust pipeline should include separation of training and testing data, external validation between institutions, calibration assessment, discrimination and clinical utility evaluation, and, if possible, temporal validation in prospectively recruited cohort of patients.

However, the application of AI to clinical neuro-oncology practice entails more than just high statistical accuracy. Models need to exhibit robustness, appropriate calibration, data drift resistance, good reporting standards, and integration into current clinical processes. The issues of regulation, privacy, biases, representative patient data, and clinician education also need to be addressed. Multisite prospective research, and eventually pragmatic clinical trials, are needed to confirm whether decision support based on artificial intelligence adds value to current clinical and pathological methods. Currently, it is widely noted that insufficient prospective validation, variation between sites, interpretability, and other factors continue to constitute key hurdles in routine adoption of AI-guided GBM strategies (Abuhassan et al., 2026).

In the context of the microbiome-mitochondria-GBM hypothesis, the role of AI is therefore not only as a predictive tool, but rather as an integrator of biological data that generates testable hypotheses and creates clinically meaningful risk models. Integrative studies that utilize longitudinal measurements of the microbiome and metabolomics alongside mitochondria phenotyping, single cell and spatial biology, imaging, treatment, and outcome data might lay the groundwork for these kinds of models. It is important, however, to link computational predictions with experimental and clinical confirmation to distinguish associations from biological causality.

### 8.4 Experimental models and clinical translation

Progress in technologies for profiling will have to be matched with experimental systems allowing to test causality. Traditional GBM cell lines and animal models are still a powerful tool for mechanistic studies, however they fail to mimic cellular heterogeneity, spatial patterning, immune system of humans, and interactions with microbiome in patients. Patient-derived GBM organoids, brain organoids, organoid-immune co-cultures, and humanized microbiome mouse models are among the advanced systems available for investigation of these phenomena (Yadav and Purow, 2024; Cox-Holmes et al., 2024; Thomas and Rahman, 2025). One type of such systems may be useful for testing whether patient-associated microbiomes are capable of altering tumor biology in a causal way. In one study, there were significant differences in the microbiome of gut in glioma patients versus healthy controls, and gliomas developed more quickly in mice transplanted with microbiomes of HGG patients (HGG-FMT), compared to mice transplanted with microbiomes from healthy controls (HC-FMT). HGG-FMT mice had higher numbers of Eisenbergiella, Mailhella, and Merdimonas, while HC-FMT mice were enriched in Limosilactobacillus and Anaerospora (Wang et al., 2025). This provides an experimental evidence for a connection between gut microbiome and glioma progression, but not the specific mitochondrial mechanisms causing the observed phenomenon.

Patient-derived organoids and xenografts may be useful as alternative platforms for testing of microbial metabolites, mitochondrial inhibitors, and combination treatments on genetically/phenotypically relevant tumors (Ishahak et al., 2025). The addition of immune cells and (if possible) microbiome-derived metabolites to such models would further enhance their capabilities for testing the proposed microbiome-mitochondria-immune axis. A key future task therefore would be the development of multi-compartment experimental systems that connect the gut, circulation, and brain-tumor microenvironment. Such systems will allow to manipulate the defined microbial community/metabolites, then measure mitochondrial function, tumor metabolism, immune-cell states, and response to treatments. This will allow to distinguish correlation from causality and determine which components of the microbiome-mitochondria axis can be therapeutically targeted.

A causal experimental design should consist of the manipulative approach with regards to potential microbial taxa and/or metabolites combined with a direct observation of tumor and mitochondrial phenotypes. The patient-derived GBM organoids and orthotopic xenografts can be exposed to different metabolites, conditioned medium from the microbiome, or selected microbial communities and their effects on tumor proliferation, invasion, stemness, therapy response, and immune cell states can be studied. Causation can be established via perturbation and rescue experiments where candidate microbes or their signals can be manipulated, and the resultant phenotype can be compared to control.

The metabolomic profiling approach should be utilized in order to establish how the modulation of the microbiome affects the microbial- and host-originated metabolic pathways, with the use of targeted and untargeted techniques, when necessary (Costantini et al., 2025; Kakde et al., 2026). Alongside with these studies, the functional assays regarding mitochondria can be performed, which will involve oxygen consumption and extracellular acidification assays, the levels of ATP production, mitochondrial membrane potential, production of mitochondrial ROS, activities of respiratory chain enzymes, mtDNA integrity, mitochondrial morphology, and markers of mitophagy, such as PINK1 and Parkin (Picca et al., 2023; Abrigo et al., 2023). In combination with mitochondrial phenotyping, the transcriptional, proteomic, and even spatial profiling can reveal whether the candidate microbial signal appears first and induces the reproducible mitochondrial phenotype, and then the latter influences GBM progression or therapeutic response. A longitudinal and mechanistic approach will provide an additional level of causation by determining how the changes in the microbiome and its metabolites, mitochondrial phenotypes, tumor phenotypes, and treatment response occur over time. Perturbation-rescue experiments that test whether blocking a candidate mitochondrial pathway prevents the tumor phenotype due to a defined microbial metabolite or vice versa can reveal the causative link between the two.

While these advances promise much progress, there are many translational challenges ahead. The composition of the microbiome depends on the patient’s diet, geographic region, medications used, treatment history, age, and other features of the host organism. Experimental systems might also significantly diverge from human biology, and variations in the methods of sample collection, sequencing, metabolomics and mitochondrial assays could limit reproducibility. Therefore, clinical studies combining the information on microbiome composition, its metabolites, mitochondrial phenotypes, immune states, tumor biology, and treatment response will be needed.

### 8.5 Evidence gaps, methodological limitations, and translational challenges

#### 8.5.1 Lack of direct human mechanistic evidence

In all the sections of this review, GBM-specific data will be defined as information that is based on human GBM populations, GBM-derived material, glioma-specific cells or animal models, and microbiome-related studies on gliomas; information from other cancer types, neurological diseases, intestines, or other cell models is regarded as complementary or extrapolated data rather than the demonstration of the mechanism in GBM. While there is growing interest in the role of the gut microbiome in GBM, there are still insufficient mechanistic data in humans. The associations have been observed in human studies among the gut microbiome composition, gut microbial metabolites, immune properties, tumor attributes, and clinical outcomes. However, relatively few studies have assessed these components in the same patients along with mitochondrial function and tumor metabolism (Ganesh et al., 2025; Toumazi et al., 2025; Weathers et al., 2025). Most of the microbiome-mitochondria connection postulates are based on additional evidence coming from microbiome-host metabolic interactions, neurological disorders, other forms of cancer, and experiments (Green et al., 2024; Koynova-Tenchov, 2026). There are no sufficient data to prove the fact that specific modifications in the human gut microbiome can directly affect the mitochondrial functioning in GBM and associated immune cells. Thus, the microbiome-mitochondria-GBM development axis has to be viewed as an emerging mechanism.

#### 8.5.2 Confounding factors in microbiome studies

The interpretation of microbiome findings is challenging due to considerable individual and temporal differences in the composition of the microbes. The diet, place of living, age, lifestyle, coexisting diseases, medication use, antibiotic use, use of corticosteroids and anti-cancer therapies, among other patient factors, can affect the presence and function of microorganisms (Hajjar et al., 2026; Toumazi et al., 2025). For GBM, the treatment-related alterations may also impact the composition of the gut microbiome and metabolome, complicating the assessment of whether the identified microbiome pattern pre-dates the disease or results from the disease process itself or from the effects of treatment (Ganesh et al., 2025). Variations in the techniques used for sampling, processing, DNA isolation, sequencing, taxonomic and metabolomic analyses can also add to the inconsistency between different studies.

#### 8.5.3 Difficulty distinguishing correlation from causation

The majority of the current human microbiome studies available are based on observations and hence cannot easily determine whether the observed change in the microbiome plays any role in the tumor physiology or is a result of either the disease itself, treatment, diet, or systemic metabolism (Ganesh et al., 2025; Toumazi et al., 2025). A relationship observed between a metabolite and mitochondrial activity likewise cannot prove that the metabolite is the reason for the observed change in mitochondrial activity. Experiments where the gut microorganisms are manipulated have revealed an effect of the microbial communities on glioma progression and responsiveness; nevertheless, such experiments in themselves do not establish the mitochondrial mechanism behind it (Hou et al., 2023; Wang et al., 2025). Causality determination will hence need to be performed via the manipulation of specific microorganisms or metabolites coupled with measurement of mitochondrial respiration, membrane potential, ROS formation, mtDNA integrity, mitochondrial dynamics, mitophagy, and the resulting phenotype.

#### 8.5.4 Limitations of longitudinal multi-omics evidence

Multi-time point analyses of the microbiome, metabolomics, mitochondria, immune system, tumor, imaging, and clinical data would be likely to yield a more informative picture of disease progression and treatment response than that from single-time point analyses. The recent human studies are beginning to make use of the integration of the microbiome profile and tumor immunity, DNA-repair activities, and clinical data, demonstrating the possibility of this approach (Dono et al., 2025). Nevertheless, the longitudinal multi-omics studies are methodologically complicated tasks. The composition of the microbiome and the levels of metabolites change relatively quickly, whereas tumor development, treatment, immune system reactions, and mitochondrial phenotypes can take place at different time-scales. The issues of missing data, variable sampling intervals, batch effects, high dimensionality, and compositional nature of the microbiome are additional complicating factors (Jiang et al., 2025; Kakde et al., 2026). Thus, the presence of longitudinal relationships cannot be considered as proof of causality. Sampling within predefined temporal windows and proper statistical analysis would be necessary in order to determine whether the microbiome-mitochondrial signatures are consistent predictors of progression or treatment response.

#### 8.5.5 Limitations of gut-brain-tumor experimental models

Experimental models are vital for causality testing but fail to capture all complexities of the human gut-brain-tumor interactions. Standard GBM cell lines are devoid of the heterogeneity, organization, interaction with the immune system, and metabolic variety of patient tumors, while conventional animal models differ from human beings in terms of microbiome, immune system physiology, intestinal microenvironment, and host metabolism (Yadav and Purow, 2024; Thomas and Rahman, 2025). Humanized microbiome mice, patient-derived xenografts, GBM organoids, brain organoids, and organoid-immune co-cultures offer increasingly more complex alternatives to conventional models but still manage to capture only certain parts of the human body (Cox-Holmes et al., 2024; Thomas and Rahman, 2025). In particular, recent humanized microbiome studies found out that feces of the patients with high-grade gliomas may affect gliomas’ development in experimental conditions, thus proving the existence of gut microbiome-glioma connection on the experimental level, even though the exact mitochondrial mechanisms have yet to be clarified (Wang et al., 2025). Patient-derived and genetically engineered organoids might also become valuable tools in investigation of tumor heterogeneity and individual response (Ishahak et al., 2025). Consequently, future experimental platforms should combine defined microbial consortia or microbiota-derived metabolites and patient-derived tumor models with mitochondrial phenotyping in order to identify the causal and therapeutically valuable elements of the proposed microbiome-mitochondria axis.

## 9 Conclusion and future perspective

Recent data suggest a wider scope for neuro-oncology involving brain tumors’ responses to genetic and microenvironmental factors but also to systemic metabolic, immune, and microbiome stimuli. Gut microbiota can affect immunity, metabolism, barrier function, and mitochondrial processes via microbial metabolites, and mitochondrial dysfunctions can, in turn, impact gut microbiome dynamics. All of these aspects can affect brain tumors’ adaptation, stemness, invasiveness, hypoxia, immune escape, treatment resistance, and relapse, but there is a lack of data on microbiome’s impact on mitochondrial signaling in brain tumors in humans.

This gap in our knowledge requires us to shift our focus from associations between bacteria and brain tumors to causal studies. Longitudinal assessment of the microbiome, metabolomics, mitochondrial processes, tumor metabolism, immune phenotypes, treatment outcomes, combined with patient-derived models and xenografts and humanized gut microbiome models, will be critical for establishing causality and validating biomarkers.

As far as therapy is concerned, we need to strive for selectively targeting maladaptive interplay between the microbiome, microbial metabolites, mitochondria, tumor metabolism, and immunoregulation, instead of manipulating either of these elements nonspecifically. Future therapeutic interventions can use a combination of microbiome modulation, metabolic therapy, mitochondria-targeted treatment, and immunomodulation depending on tumor characteristics. In summary, the microbiome-mitochondria interface should currently be considered as a promising mechanism but not a proven therapeutic target yet.
